# Green-synthesized reduced graphene oxide@chitosan beads for the removal of polycyclic aromatic hydrocarbons

**DOI:** 10.1007/s11356-025-37364-6

**Published:** 2026-01-21

**Authors:** Marina Barbosa de Farias, Albertina Gonçalves Rios, Alexandre Filipe Porfírio Ferreira, Patrícia Prediger, Melissa Gurgel Adeodato Vieira

**Affiliations:** 1 School of Chemical Engineering, Universidade Estadual de Campinas, Albert Einstein Av. 500, Campinas, São Paulo 13083-852 Brazil; 2 LSRE-LCM – Laboratory of Separation and Reaction Engineering - Laboratory of Catalysis and Materials, Faculty of Engineering, Universitdade Do Porto, Rua Dr Roberto Frias S/N, 4200-465 Porto, Portugal; 3 ALiCE – Associate Laboratory in Chemical Engineering, Faculty of Engineering, Universitdade Do Porto, Rua Dr Roberto Frias S/N, 4200-465 Porto, Portugal; 4 School of Technology, Universidade Estadual de Campinas, Limeira, São Paulo 13484-332 Brazil

**Keywords:** Polycyclic aromatic hydrocarbons, Produced water, Green synthesis, Biopolymer, Adsorption, Reduced graphene oxide

## Abstract

Reduced graphene oxide and iron nanoparticles were prepared through a one-pot green route with eucalyptus leaf extract as a reducing agent and immobilized into chitosan beads (m-rGO@CS). In this study, the adsorption performance of these m-rGO@CS beads towards anthracene (ANT) and fluoranthene (FLT) was assessed through adsorption equilibrium, adsorption kinetics and thermodynamic analyses. An adsorbent dosage of 0.5 g/L and pH 8 was determined as the best operating conditions through a full-factorial design. The adsorption kinetic data of the contaminants were well-described by the pseudo-second-order model and indicated that the process might involve film and intraparticle diffusions as the main controlling steps. The equilibrium time for ANT and FLT adsorption was 360 and 1320 min, respectively. The Sips isothermal model best fitted the equilibrium data. The maximum removal capacity towards ANT and FLT was 16.97 and 12.19 mg/g, respectively. The thermodynamic parameters showed that the ANT and FLT adsorption processes are spontaneous and exothermic. The regeneration tests indicated that the material presented a decrease of 2% and 8% in the adsorption capacity of ANT and FLT, respectively, after three adsorption–desorption cycles.

**Graphical Abstract:**

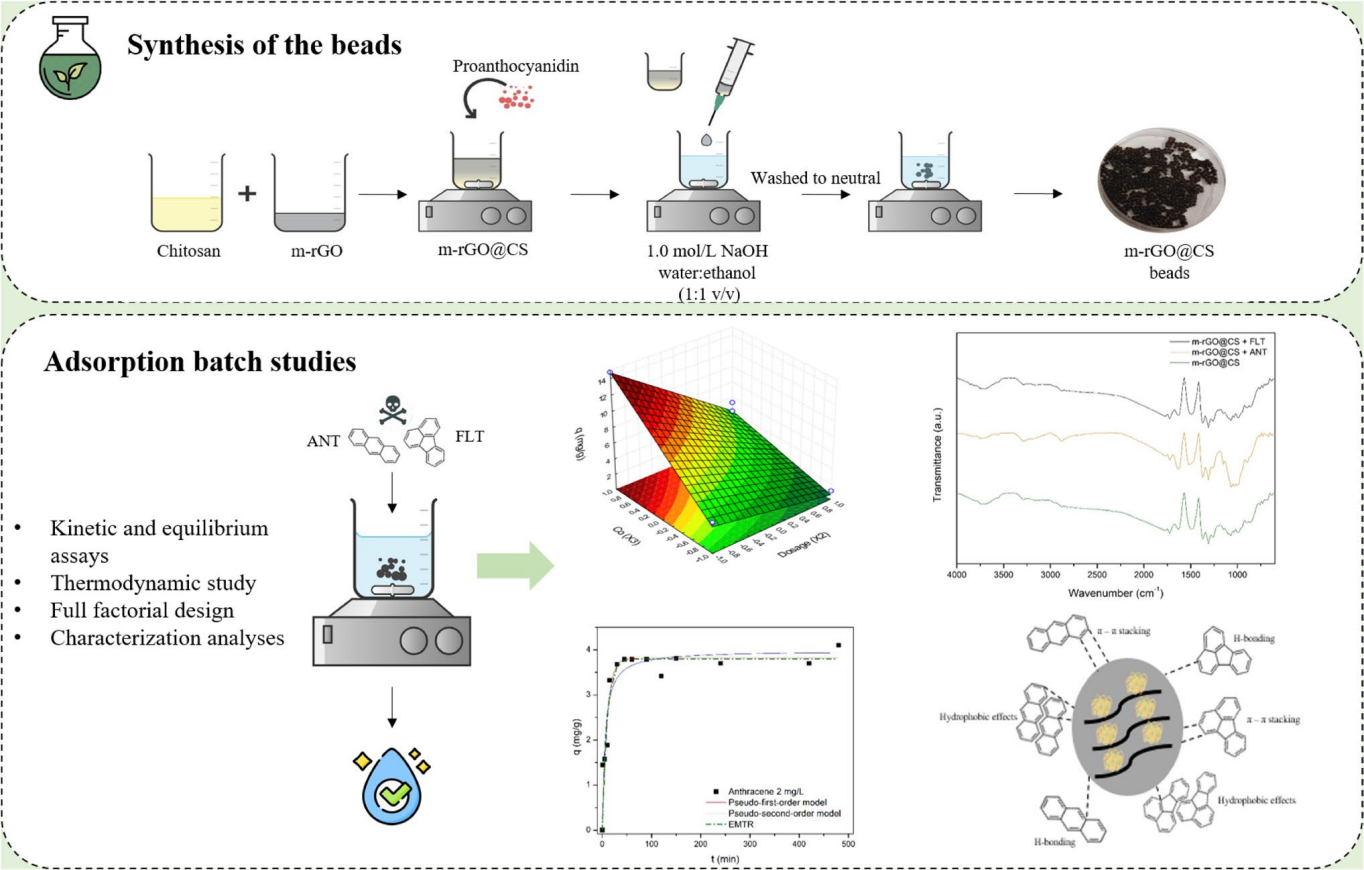

**Supplementary Information:**

The online version contains supplementary material available at 10.1007/s11356-025-37364-6.

## Introduction

The massive volume of effluents generated by the petroleum and gas industry is a source of great concern. Produced water (PW) is one of the largest effluents from this sector and refers to the water that comes out from the reservoirs during oil and gas production. Its discharge into the environment is of great concern, as its complex composition and large volume classify it as a significant source of contamination of water bodies (Salem and Thiemann 2022). The main characteristics of PW can vary considerably depending on the location and age of the well, the geological formation, and the types of exploration and production processes. In general, the primary components of PW include polycyclic aromatic hydrocarbons (PAHs); benzene, toluene, ethylbenzene, and xylene (BTEX); phenol; carboxylic acids; dissolved minerals; and treatment chemicals (Al-Ghouti et al. 2019). Aromatic hydrocarbons and some phenols are particularly concerning due to their bioaccumulation potential (Neff et al. 2011). Therefore, properly managing produced water is critical to environmental sustainability; it also contributes to the cost-effectiveness of oil and gas production because this waste stream can be reused.

A combination of mechanical and chemical processes is usually effective in removing oil, grease, and volatile compounds. However, eliminating dispersed solids and diluting toxic compounds demands further treatment for the PW to ensure compliance with the guidelines for its disposal or reuse (Liang et al. 2018). Adsorption is one of the advanced separation methods that has been efficiently used for environmental applications. It is a simple, flexible and cost-effective technology that allows the reuse of the adsorbent material after proper regeneration (Özbay et al. 2013). The efficiency and quality of the process depend directly on the choice of the adsorbent; therefore, efforts have been made to develop materials with desired structures that follow essential features, namely great adsorption capacity, good cost-effectiveness and environmental friendliness (Al-Kaabi et al. 2021).

Graphene-based materials have attracted significant research interest in environmental remediation areas. For example, graphene, graphene oxide (GO) and reduced graphene oxide (rGO) can efficiently remove organic and inorganic pollutants from aqueous media. rGO has a one-atom-thick structure usually prepared by reducing GO via chemical, electrochemical or thermal reduction. It differs from pristine graphene due to residual functional groups and surface defects, allowing modifications in surface chemistry for specific applications and expanding its applicability (Shahdeo et al. 2020). One of the disadvantages of rGO compared to graphene is the tendency for sheets to aggregate and restack. This drawback can be overcome by the synthesis of 3D-graphene networks and the production of graphene-hybrids and composites by combining graphene-based material with others, such as ceramic, carbon, metal and polymers (Hashempour et al. 2023).

For instance, chitosan (CS), a natural polymer with good biodegradability, biocompatibility, and non-toxicity, has shown efficient adsorption capacity. Its properties make it suitable for biological, agricultural and environmental applications (Aizat and Aziz 2019). CS is an outstanding biopolymer for the development of composites since it provides plenty of functional moieties for efficient adsorption. Combining CS with reduced graphene oxide can enhance its mechanical strength, temperature sensitivity and adsorption performance (Pan et al. 2011). The literature shows that hybrid rGO/chitosan materials have been applied in several areas, such as drug loading and delivery, electrochemical applications, food packaging, and bone tissue engineering (Olalekan et al. 2021). The use of natural agents, such as plant extracts, yeast, fungi, bacteria, proteins, and carbohydrates, provides an environmentally friendly approach to developing adsorbent materials, reducing the application of hazardous chemicals (Queiroz et al. 2023). Green materials contain compounds that can act as reducing, capping, and crosslinking agents.

The present work introduces a novel chitosan/reduced graphene oxide composite (m-rGO@CS) synthesized through a green reduction route employing *Eucalyptus* leaf extract and proanthocyanidins**.** The *Eucalyptus* leaf extract was strategically chosen owing to its rich composition of phenolics, flavonoids, and terpenoids, which endow it with strong reducing power and excellent capping ability (Jin et al. 2018). These phytochemicals present in the extract and proanthocyanidins act as natural reducing and crosslinking agents, enabling the efficient reduction of graphene oxide and improving the structural stability and surface functionality of the composite (Wang et al. 2019; Xiao et al. 2019; Queiroz et al. 2022a). Therefore, the use of *Eucalyptus* extract could confer distinct chemical and morphological advantages compared with other green synthesis methods, resulting in an m-rGO@CS composite with enhanced surface functionality and adsorption performance. This eco-friendly and more cost-effective synthesis decreases the use of hazardous chemical reducers, such as hydrazine, aligning with sustainable material development principles (Queiroz et al. 2022b). The resulting composite exhibits enhanced dispersion, abundant active sites, and easy separability, offering practical advantages for water treatment applications compared to conventional adsorbents.

The obtained m-rGO@CS beads were evaluated as adsorbents for the uptake of polycyclic hydrocarbons, namely anthracene (ANT) and fluoranthene (FLT), which are good representative PW contaminants. First, a full-factorial experimental design was applied to optimize the operating conditions. Then, kinetic, equilibrium and thermodynamic studies were conducted to understand the nature and possible mechanisms of the process. In addition, batch regeneration assays were performed to assess the reuse potential of the materials. The synthesized adsorbents were also characterized after the adsorption to get further insights regarding their structure and functionalities, and possible adsorption mechanisms.

## Methodology

### Development of m-rGO@CS beads through green synthesis

Graphene oxide (GO) and FeCl_3_ were simultaneously reduced in a single green process reported by Xiao et al. (2019), which used *Eucalyptus* leaf extract as the reducing agent. Concisely, a mixture of GO suspension (0.5 g/L) and FeCl_3_.6H_2_O (0.01 M) (1:1 v/v) was sonicated for 30 min. The *Eucalyptus* leaf extract was prepared by adding the leaves to ultrapure water (0.06 g/mL), the system was heated for one hour at 80 °C, filtered and stored. Then, the *Eucalyptus* leaf extract was added to the mixture (2:1 v/v), which was put in a water bath (80 °C) and submitted to constant agitation for eight hours. The obtained product (m-rGO) was filtered, washed with water and methanol, and stored.

**Fig. 1:**
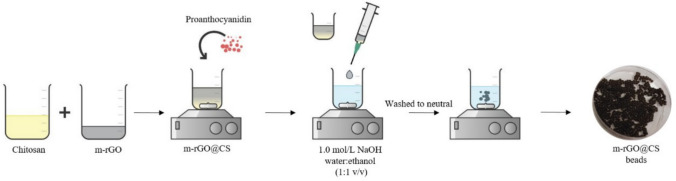
Schematic representation of the m-rGO@CS beads green synthesis

The detailed synthesis of green beads (m-rGO@CS) using m-rGO is described in a previous study conducted by de Farias et al. (2024). Figure 1 displays its schematic representation. Briefly, a chitosan solution (3% w/v) was prepared by dissolving it in 2% acetic acid. The m-rGO (7% w/v) was added to the CS solution and stirred for 1 h. Then, proanthocyanidin (10% w/v) was added to the system, which was subjected to constant stirring for another hour. Drops of the obtained product were added to a 1.0 mol/L NaOH water–ethanol solution (1:1 v/v) at 350 rpm with a syringe. The beads were inserted in a new NaOH water–ethanol solution and submitted to continuous agitation for 30 min. Finally, they were washed with ethanol and distilled water until a neutral pH solution was obtained and dried at room temperature for 48 h.

### Characterization analysis

The analyses displayed in Table 1 assessed the physicochemical properties of the m-rGO@CS beads.

**Table 1:** Characterization analyses of contaminated m-rGO@CS

Analysis	Equipment	Parameters
Thermal analysis (TG/DTG)	DTG-60, Shimadzu, Japan	N_2_ atmosphere, 50 mL.min^−1^ flux, range 25 °C to 1000 °C, heating rate of 10 °C.min^−1^
X-ray photoelectron spectroscopy (XPS)	K-alpha XPS Thermo Scientific	Al Kα (h ν = 1486 eV) radiation, 10^−9^ Pa. Spot Size 300 µm; Pass Energy 100.0 eV; Energy Step Size 1.000 eV; Dwell Time 10 ms
X-ray diffraction (XRD)	Shimadzu XRD7000	Cu Kα radiation with λ = 0.154 nm, operating at 40 kV and 30 mA, at 25 °C
Fourier transform infrared spectroscopy (FTIR)	Thermo Scientific equipment (Nicolet 6700, Madison/USA)	KBr method, range 4000–400 cm^−1^, resolution 4 cm^−1^, and scan 32
Nitrogen adsorption–desorption	Micromeritrics (ASAP 2020 Plus, Norcross, EUA)	Sample treatment at 80 °C until constant pressure is reached (~ 6 μmHg, ~ 30 h, degas < 20 μmHg/min)

### Full-factorial design

The influence of pH, adsorbent dosage (g/L), and initial solution concentration (mg/L) on the adsorption capacity of the beads was assessed through a 2^3^-full-factorial design. The chosen factors and levels are described in Table S1 of the Supplementary Material. For this, batch experiments were carried out in beakers containing a pre-determined mass of m-rGO@CS and 10 mL of each contaminant solution under constant stirring in an orbital shaker for 24 h at 25 °C. After the set time, aliquots were collected, and the supernatant solution concentration of anthracene and fluoranthene was determined in a UV–Vis spectrophotometer (Shimadzu/UV-1900) at λ of 251 and 236 nm, respectively. The adsorption capacity was determined following Eq. (1). Statistica® software was applied to evaluate the experimental data to determine the best conditions for further adsorption assays.

1 $${q}_{t}=\frac{\left({C}_{o}-{C}_{t}\right)V}{m}$$

where q_t_ is the adsorption capacity (mg/g), C_o_ is the initial concentration of the contaminant solution (mg/L), C_t_ is the concentration of the solution at time t (mg/L), V is the volume of the solution (L), and m is the mass of the adsorbent (g).

### Kinetic adsorption assays

The kinetic assays were conducted in beakers containing an adsorbent dosage of 0.5 g/L. Initial concentrations of C_o_ = 2, 3.5 and 5 mg/L were adopted for both ANT and FLT. The tests were submitted to constant stirring at 25 °C and 200 rpm. Blank tests were also done to avoid any interference. Aliquots were collected at pre-determined times; the residual concentration of each adsorbent was determined using a UV–Vis spectrophotometer, which was used to calculate the adsorption capacity following Eq. (1).

The models of Pseudo-first order (Lagergren 1898), Pseudo-second order (Ho and Mckay 1998), intraparticle diffusion (IPD) (Weber and Morris 1963), external mass transfer resistance (EMTR) (Puranik et al. 1999), Boyd’s (Boyd et al. 1947), Linear driving force (LDF) (Glueckauf and Coates 1947), Fickian diffusion (FD) (Vermeulen 1953) were adjusted to the experimental data using OriginPro® 8 and Maple®17 software. The equations of kinetic models are detailed in the Supplementary Material (Table S2).

### Adsorption equilibrium assays

The equilibrium study was conducted on a rotary shaker with the initial solution concentration of ANT and FLT ranging from 2 to 8 mg/L, pH 8 and a fixed dosage of 0.5 g/L. The assays were stirred at 200 rpm for 24 h at three constant temperatures: 25, 35 and 45 °C. After the determined time, aliquots of the tests were collected, and the residual concentrations were calculated. The isothermal models of Langmuir (1918), Freundlich (1906) and Sips (1948) were adjusted to the experimental data using OriginPro® 8 software. The equations of equilibrium models are detailed in the Supplementary Material (Table S3).

### Thermodynamic study

The equilibrium data were used to estimate the thermodynamic parameters of Gibbs energy change (∆*G*, kJ/mol), enthalpy change (∆*H*, kJ/mol) and entropy change (∆*S*, J/mol/K) by Eq. (2).

2 $$\ln\;K_c=\frac{-\Delta G}{RT}=\frac{\Delta S}R-\frac{\Delta H}{RT}$$

where R is the universal gas constant (8.314 J/mol.K), T is the temperature (K), and K_c_ is the thermodynamic equilibrium constant. Several approaches have been adopted to determine the constant K_c_. Herein, K_c_ was obtained from the slope of q_e_ versus C_e_. As suggested by Milonjic (2007), the dimensionless K_c_ was obtained by multiplying the constant by 1000. The slope and the intercept of lnK_c_ versus 1/T were used to estimate enthalpy and entropy changes, respectively.

The isosteric enthalpy of adsorption (ΔH_st_) was determined by the Clausius–Clayperon equation (Eq. (3)). The isosteres of the ANT and FLT adsorption onto the m-rGO@CS beads were determined at different fixed values of q_e_ at 25 °C, 35 °C and 45 °C.

3 $$ln\;C_e=\frac{{\Delta H}_{st}}R\left(\frac1T\right)+C$$

### Simplified batch design

The equilibrium data were used to predict the design of a batch adsorption system. The amount of adsorbent necessary to achieve particular ANT and FLT reduction efficiencies was determined through a mass balance using the Sips equilibrium model, as given in Eq. (4).

4 $$m=\frac{V\left({C}_{o}-{C}_{e}\right)}{\frac{{q}_{s}{\left({K}_{s}{C}_{e}\right)}^{n}}{1+{\left({K}_{s}{C}_{e}\right)}^{n}}}$$

### Regeneration of m-rGO@CS beads

The desorption assays and regeneration analysis were conducted following a methodology similar to the previous batch tests. The contaminated adsorbent (0.5 g/L), after 12 h of adsorption, was washed with ultrapure water to remove the excess PAH solution on the bead’s surface and put into beakers with ethanol. The desorption assays were stirred at 200 rpm for 8 h, at 25 °C, to ensure maximum removal. Ethanol was chosen as eluent based on previous works of Queiroz et al. (2023), who used similar materials. After the determined time, the supernatant of the desorption assay was collected, and the desorbed concentration was calculated (Eq. (5)). The regenerated beads were washed with ultrapure water and used in three consecutive adsorption–desorption cycles.

5 $$\%Des= \frac{{C}_{d}{V}_{d}}{{q}_{e}m}*100$$

where C_d_ refers to the concentration of the contaminant after desorption, V_d_ is the volume of the eluent, q_e_ is the equilibrium adsorption capacity, and m refers to the mass of the contaminated adsorbent.

## Results and discussion

### Characterization analyses

The m-rGO@CS beads were first synthesized and characterized in a previous work by de Farias et al. (2024). Briefly, the scanning electron microscopy with energy-dispersive X-ray spectroscopy (SEM–EDS) was conducted to evaluate the morphology and elemental composition of the beads. The SEM images showed that the synthesized m-rGO@CS beads have an elliptical shape and a somewhat smooth surface. The morphology of m-rGO@CS might be associated with the method in which the chitosan, reduced graphene oxide, and iron solution were manually dropped into NaOH solution to form the beads, and also the drying method of the composites. The EDS mapping of the surface’s elements showed the predominance of carbon (46%) and oxygen (44.6%), as expected, along with nitrogen (9.2%) and traces of iron (0.2%) (de Farias et al. 2024).

The zeta potential analysis was performed between pH 4 and 10. Even though chitosan is a cationic biopolymer, the m-rGO@CS beads displayed an electronegative nature as the pH of the solution increased. At pH 4, the beads showed a zeta potential of + 4.39 mV, possibly due to the protonation of the chitosan’s amine groups. At pH 10, the zeta potential was − 8.75 mV (de Farias et al. 2024). To extensively comprehend the nature and properties of the m-rGO@CS beads, further characterization analyses were conducted and presented in this section.

#### Nitrogen adsorption–desorption (BET analysis)

Nitrogen adsorption–desorption analysis showed that the m-rGO@CS composite possesses a very low BET surface area (0.16 m^2^ g⁻^1^) and exhibits a Type IV isotherm with an H3 hysteresis loop (see Supplementary Material), indicating a mesoporous solid (Thommes et al. 2015). Although the Barrett-Joyner-Halenda (BJH) pore-size distribution shows mesopores in the 2–8 nm range, larger than the molecular sizes of anthracene and fluoranthene, the negligible microporosity and the very low total pore volume (≈1.6 × 10⁻^4^ cm^3^ g⁻^1^) further confirm the limited textural development of the material. These results should be interpreted with caution, however, as the BET region in this analysis presented a poor linear fit and a low C constant, reflecting weak N₂–surface interactions. Despite the restricted textural development, the composite provides localized graphene-like aromatic domains capable of strong interactions with hydrophobic organic contaminants. Therefore, the adsorption of anthracene and fluoranthene is likely governed predominantly by π–π interactions between the PAH aromatic rings and the sp^2^ carbon domains of rGO, complemented by hydrophobic partitioning into the nonpolar interlayer regions formed between the partially overlapping sheets. These mechanisms are consistent with the low polarity of ANT and FLT and explain their affinity for the composite even in the absence of significant accessible surface area or micropore-driven adsorption (Cheng et al. 2019).

#### Thermal analysis

**Fig. 2:**
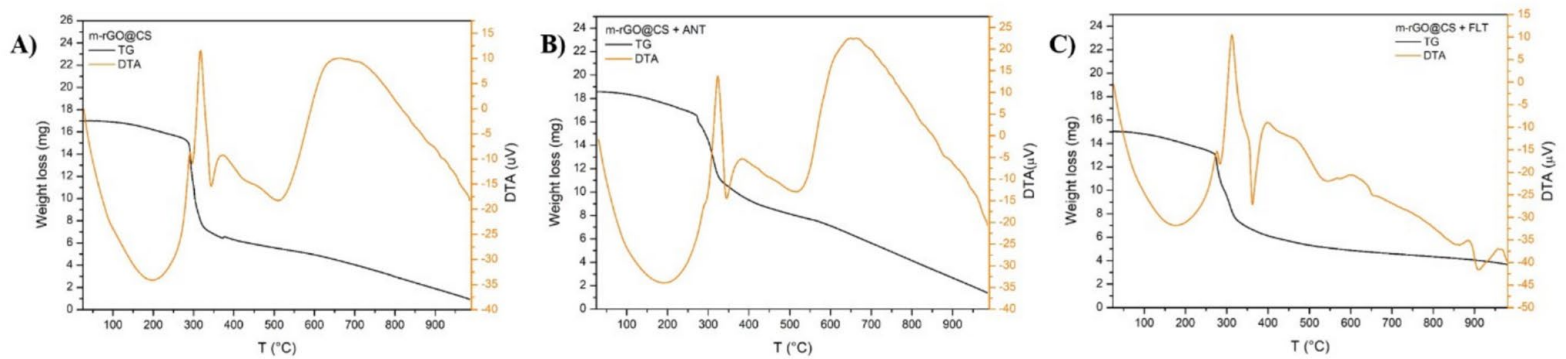
Thermogravimetric (TG) and differential thermal (DTA) analyses for (**A**) m-rGO@CS beads, (**B**) m-rGO@CS beads + anthracene and (**C**) m-rGO@CS + fluoranthene

Figure 2 displays the thermal behavior of the beads from the post-adsorption process. Both samples’ first range of weight loss occurs up to 270 °C, in which a 10% and 12% loss was observed for ANT and FLT, respectively. This initial loss may be ascribed to the dehydration of the samples due to evaporation of the surface and interlayered adsorbed water (30–100 °C) and to the initial decomposition of the biopolymer via deacetylation and breakdown of glycosidic units, which starts around 130 °C (Villar-Chavero et al. 2018). Then, a more expressive mass loss is observed from 270 °C to near 550 °C, 58.6% for ANT and 66.2% for FLT. This second loss region might refer to the pyrolysis of the biopolymer (300–470 °C) (Tovar et al. 2020), pyrolysis of rGO carbon backbone (320–550 °C) (Nayl et al. 2022) and pyrolysis of oxygenated groups of rGO and CS, as well as the decomposition of the contaminants (Tovar et al. 2020). After 750 °C, the FLT-contaminated bead is quite stable, with only 5% mass loss. Conversely, the ANT-contaminated beads lost thermal stability, as shown in Fig. 2b, a behavior similar to that of the pure beads (Fig. 2c). It is possible to observe that pre-contaminated m-rGO@CS beads exhibited lower thermal stability than the contaminated ones, as a more abrupt mass loss is noted around 350 °C (~ 60%), followed by a steady decrease. It might indicate that the adsorbate-adsorbent bond strengthens the crosslinking of the bead precursors, slowing down its thermal degradation (Patinõ-Ruiz et al. 2020).

#### XRD analysis

**Fig. 3:**
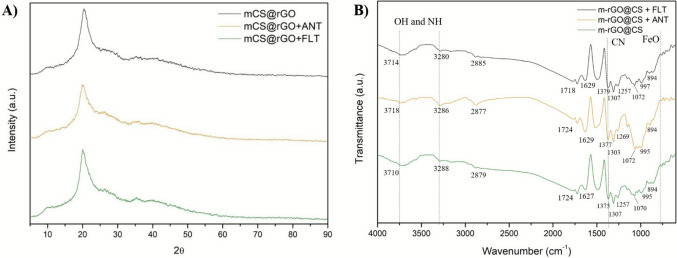
**A** Diffractogram patterns of m-rGO@CS before and after the adsorption process and **B** FTIR spectra of m-rGO@CS, m-rGO@CS + ANT and m-rGO@CS + FLT

XRD analysis evaluated the crystal structure of the m-rGO@CS synthesized before and after the adsorption process (Fig. 3a). The peaks observed at 10.1° and 20.1° are characteristic of crystalline chitosan. In addition, two distinct peaks of iron particles were observed at 35.2° and 40.5° (Queiroz et al. 2023). A broad region is observed around 2θ ≈ 25 °C, which is indicative of the presence of reduced graphene oxide in the beads (Dhanavel et al. 2020). The greater intensity of the characteristic peaks of chitosan indicates the greater concentration of this biopolymer in the beads. In addition, the presence of these diffraction angles endorses the effective formation of the composite (Dhanavel et al. 2020). It is noticeable that the adsorption process did not significantly alter the structure of the beads.

#### XPS analysis

In this work, XPS analysis was performed to determine the elemental composition of the beads before and after PAH adsorption. The broad-survey scans are depicted in the Supplementary Material. The pristine beads show the presence of C (62.7%), O (30.7%), N (6.5%), and small traces of Fe (0.1%). The variation in the proportion of these elements indicates the presence of contaminants in the beads after the process. The spectrum of ANT-contaminated beads showed only minor changes in composition (C 63.5%, O 29.2%, N 7.2%, Fe 0.1%). On the other hand, after FLT adsorption, there was a notable increase in the percentage of carbon (72.2%) and a decrease in oxygen (23.3%), nitrogen (4.4%), and iron (0.08%). This can be attributed to the presence of FLT molecules that are attached to the adsorbent surface, which is primarily composed of carbon. Furthermore, the more pronounced changes in the m-rGO@CS + FLT beads suggest that this PAH has superior interactions with the adsorbent, corroborated by its higher adsorption capacity in the kinetic and equilibrium studies (de Andrade et al. 2020).

**Fig. 4:**
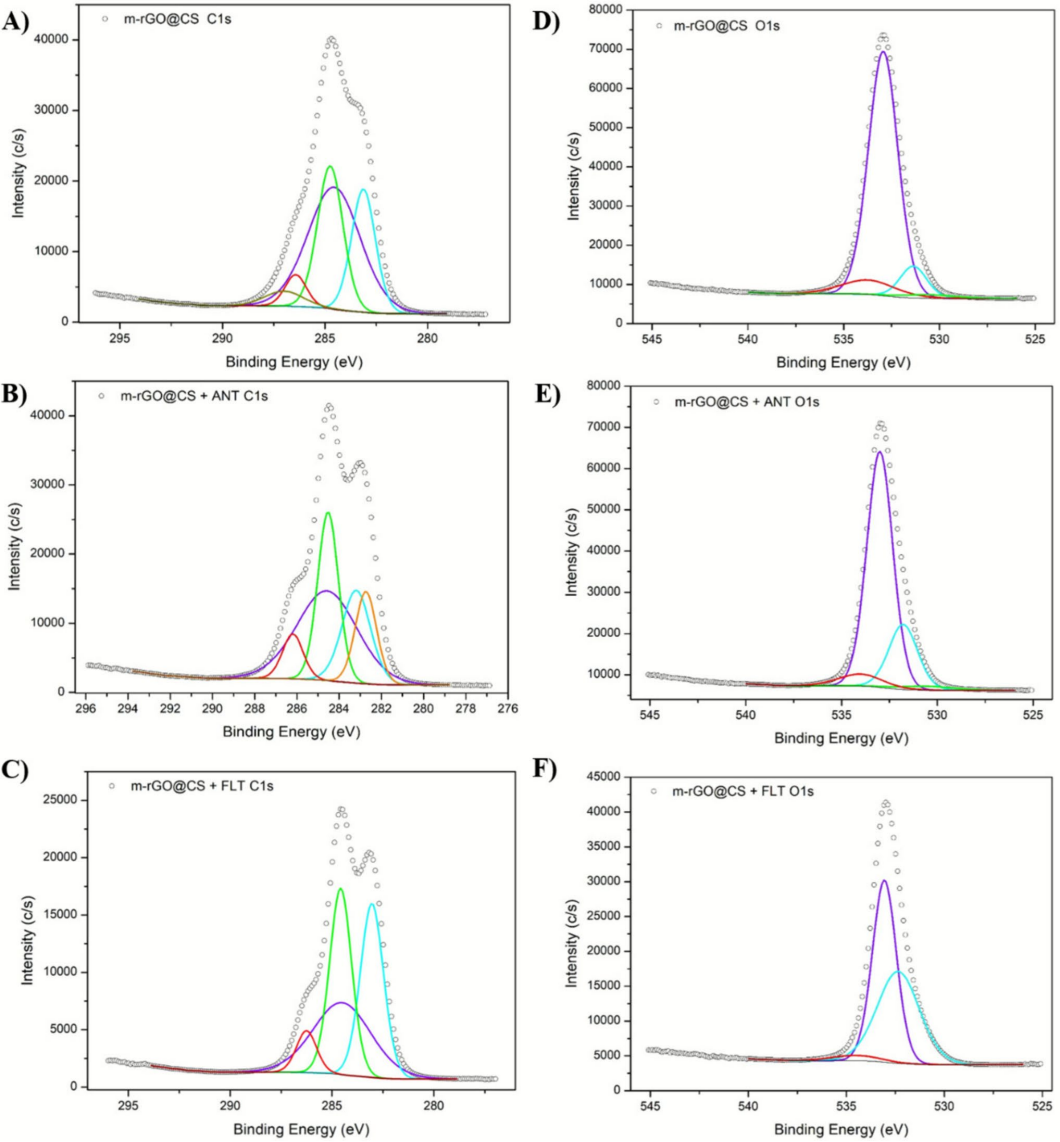
High-resolution XPS spectra of C1s (**A**–**C**) and O1s (**D**–**F**) for m-rGO@CS, m-rGO@CS + ANT, and m-rGO@CS + FLT

Full high-resolution spectra for C1s are depicted in Fig. 4A–C. In the C 1 s XPS scan, the m-rGO@CS spectrum showed peaks around 283.0, 284.7, 286.4, and 287.0 eV, which correspond to C-H (Hu et al. 2015), C–C/C-H bonds, C-O/C-N bonds, and C = O bonds, respectively (Neves et al. 2022). For the ANT-contaminated beads, the peaks centered around 283.1, 284.7, and 286.4 eV shifted to 282.8, 284.5 and 286.1 eV. The peak at 287 eV did not appear after ANT adsorption, which might indicate the participation of carbonyl groups in π-π interactions between the adsorbate and the beads (Neves et al. 2022). Similarly, for the FLT-contaminated bead, the peak related to the C = O bond did not appear in the spectrum; however, the other peaks did not show significant changes. This indicates that the adsorption of both ANT and FLT alters the bonding energy of the components. Changes in the peak centered at 286.4 eV to 286.2 and 286.6 eV after FLT and ANT uptake, respectively, might suggest the involvement of alcohols and amine groups in the adsorption (de Andrade et al. 2020).

The high-resolution O 1 s deconvolution is illustrated in Fig. 4D–F. The m-rGO@CS presented peaks around 530.6, 531.3, 532.9 and 533.7 eV, associated with Fe–O (Karthika et al. 2020), C-O/C = O, C-O/C–OH, and O-C-O bonds, respectively (Duchoslav et al. 2018). After ANT contamination, these peaks shifted to 530.7, 531.8, 533.0 and 534.0 eV. After FLT adsorption, the peaks related to Fe–O and C-O/C = O disappeared, and the peaks centered at 532.9 and 533.7 eV shifted to 532.4 and 533.1 eV, respectively. In addition, a peak appeared at 534.5 eV, which might be attributed to molecularly adsorbed water (Biniak et al. 1997). These changes are probably related to the involvement of oxygenated functions in the adsorptive mechanism. The N 1 s spectra in Supplementary Material show that m-rGO@CS has noticeable peaks at 399.7 and 401.5 eV, corresponding to C-NH_2_ and NH_3_^+^ (Queiroz et al. 2023). The 399.7 eV peak shifted to 400.3, and the 401.5 eV peak disappeared for both ANT-contaminated and FLT-contaminated beads. In addition, peaks appeared at 402.7 eV for ANT-contaminated m-rGO@CS and 402.2 eV for FLT-contaminated m-rGO@CS, which might be ascribed to the presence of oxidized N^+^ moieties or cationic N centers (Vinu et al. 2008). These results suggest that nitrogen functions might play a role in adsorption through H-bonding (Neves et al. 2022).

#### FTIR analysis

The FTIR spectra of the beads before and after the adsorption process are portrayed in Fig. 3B. The m-rGO@CS beads exhibited bands around 3710 and 3288 cm^−1^ associated with OH (Lengyel et al. 2017) and NH stretching (Mincke et al. 2019). The band at 2879 cm^−1^ and the lower-intensity band at 2930 cm^−1^ are attributed to the symmetric and asymmetric modes of C-H stretching vibrations from the CS and rGO materials. The band at 1627 cm^−1^ can be assigned to C = C stretching bonds, whereas the highlighted bands at 1473, 1375 and 1303 cm^−1^ are associated with C-N elongations of chitosan surface (Chen et al. 2011; Mincke et al. 2019). Moreover, the bands at 1070 cm^−1^ and around 600 cm^−1^ correspond to the C-O stretching and the Fe–O bond, respectively (Mekahlia and Bouzid 2009). The contaminated beads showed analogous bands to those of m-rGO@CS before the adsorption process, with slight changes in the position and intensity of some bands, which may indicate the presence of ANT and FLT. More specifically, the m-rGO@CS band shifted from 1627 cm^−1^ to 1629 cm^−1^ after both adsorption processes, which may indicate the occurrence of π- π interactions between the adsorbent and the adsorbate molecules (Neves et al. 2022).

### Full-factorial design

Experimental design is a highly applicable tool for determining the most suitable operating conditions within a given range of analysis. When applied correctly, it allows the optimization of the studied variables through a limited number of trials, thereby reducing costs. In this study, experimental designs were carried out for the adsorption of anthracene and fluoranthene onto m-rGO@CS. The influence of adsorbent dosage, initial solution concentration and pH on the adsorption capacity was assessed.

**Fig. 5:**
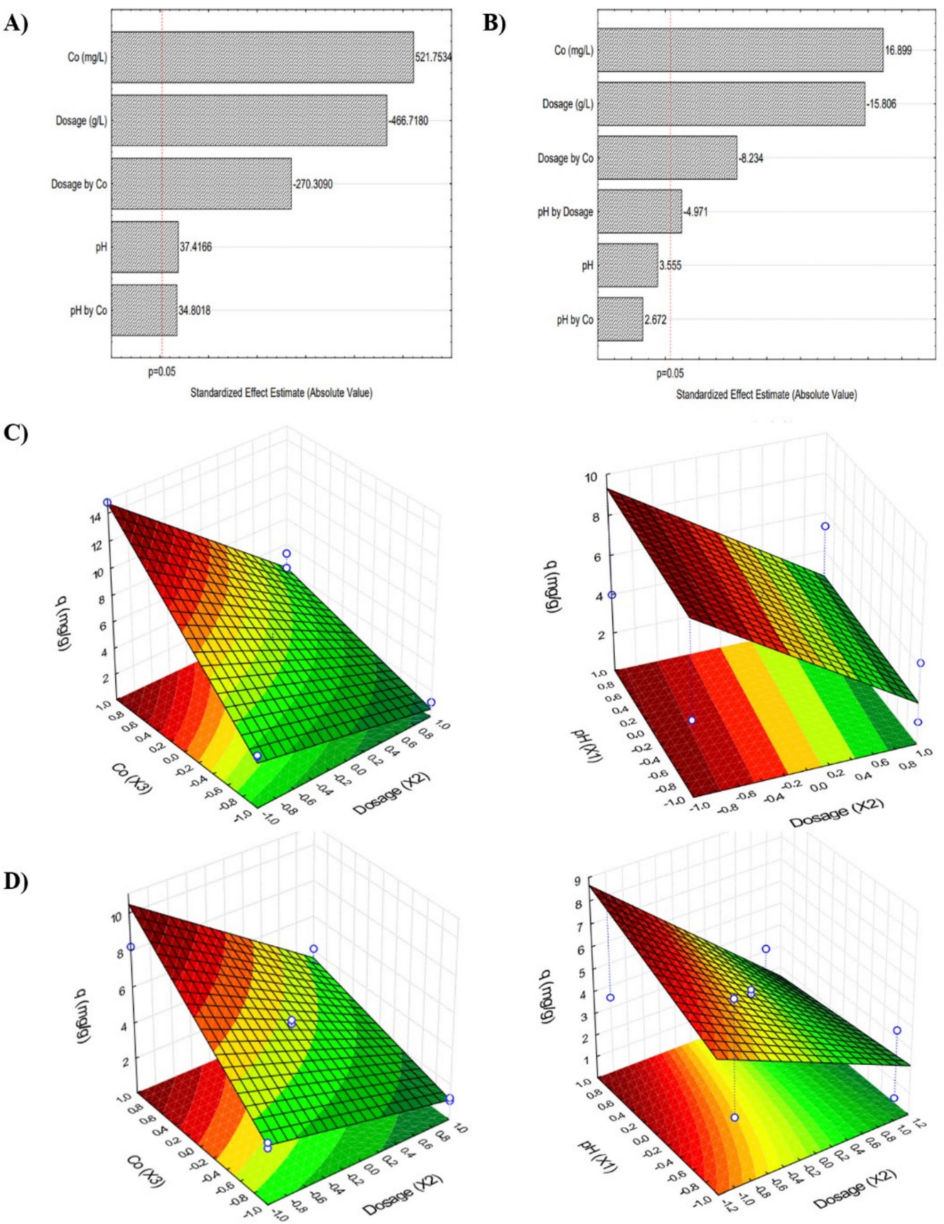
Pareto chart (absolute values) and surface plots for anthracene (**A**–**C**) and fluoranthene (**B**–**D**) adsorption

The experimental design using the green-synthesized beads for anthracene removal showed that all variables evaluated were significant within a 95% confidence level (Fig. 5a). The initial solution concentration had the most significant impact on the predicted outcome, followed by dosage and pH. Equation (6) presents the developed predictive model determined based on the ANOVA data (Supplementary Material). Since the interaction between pH and dosage was not significant, the term was removed from the model, following the backward elimination technique. The adjusted R^2^ value of 92.7%, the *p*-value below 0.05, and the randomly distributed residuals in the plot of predicted versus observed values are indicative of the statistical significance of the model.

6 $${q}_{ANTg}=5.718+0.267{x}_{1}-3.330{x}_{2}+3.722{x}_{3}+0.248{x}_{1}{x}_{3}-1.929{x}_{2}{x}_{3}$$

Among the values analyzed, the highest adsorption capacity (mg/g) predicted by the model was achieved under the conditions of 0.5 g/L, 8 mg/L, and pH 8. The Pareto chart (Fig. 5a) and the response surface plots (Fig. 5b) show that an increase in concentration and a decrease in dosage lead to higher adsorption capacity values. The negative impact of dosage, on the other hand, may indicate that an excess of adsorbent creates vacant active sites that are not adequately occupied by the adsorbate. Although the optimal pH was defined as 8, its variation is relatively insignificant when the concentration and dosage are kept constant. The desirability analysis (Supplementary Material) identified these same conditions as optimum, which estimates a removal efficiency of 96.68%.

Similarly, the experimental design using m-rGO@CS for fluoranthene uptake revealed that concentration had the most significant impact on the predicted outcome, followed by dosage. However, pH was not significant within the 95% confidence interval (Fig. 5c). Equation (7) presents the predictive model developed based on ANOVA data (see Supplementary Material). Although pH and the interaction between pH and dosage were not significant, their terms were retained in the model, as their removal decreased the overall fit. The plots of predicted versus observed values depict a random distribution, indicating the model’s satisfactory predictability. The high adjusted-R^2^ value of 90%, the *p*-value below 0.05, and the *F*-test also support the good fit of the model.

7 $${q}_{FLTg}=4.611+0.506{x}_{1}-2.251{x}_{2}+2.407{x}_{3}-0.708{x}_{1}{x}_{2}+0.381{x}_{1}{x}_{3}-1.173{x}_{2}{x}_{3}$$

Similarly, fluoranthene adsorption capacity was maximized by increasing the concentration and decreasing the adsorbent dosage. Consistent with the other analyses, the optimum operating conditions were determined to be 0.5 g/L, 8 mg/L, and pH 8.

### Adsorption kinetics

**Fig. 6:**
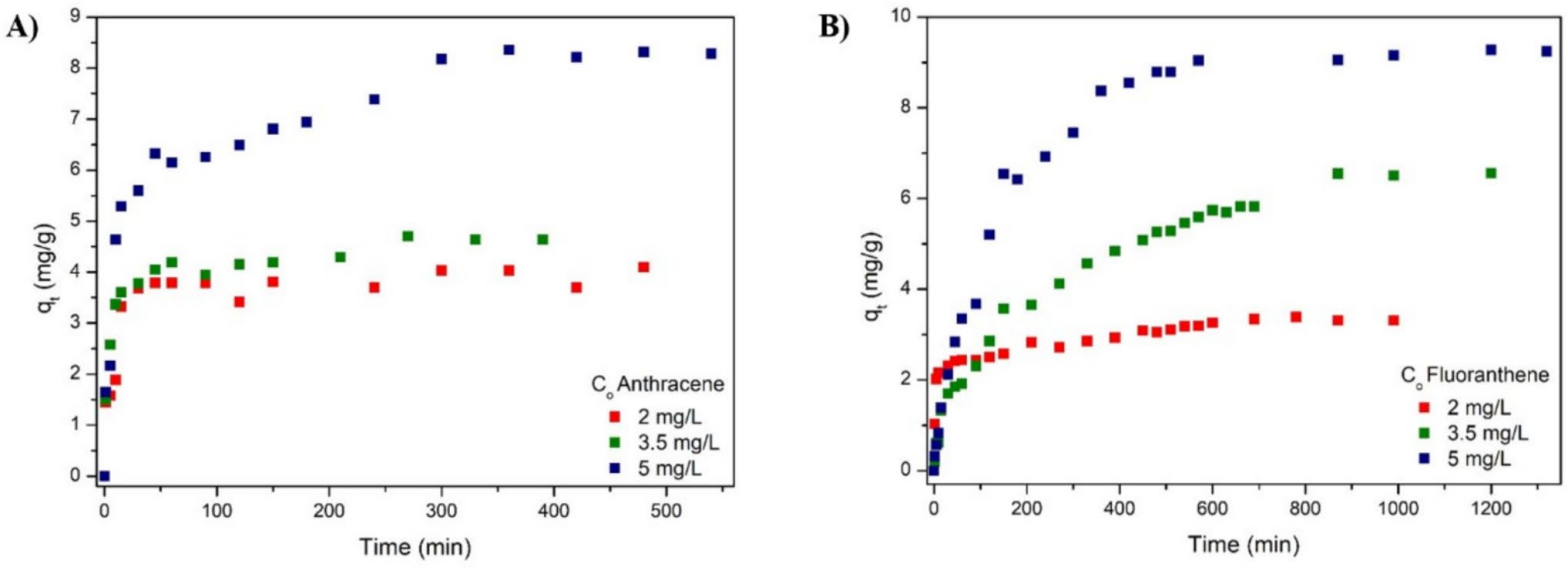
Kinetic adsorption curves of (**A**) anthracene, (**B**) fluoranthene onto m-rGO@CS

Figure 6 displays the adsorption kinetics of anthracene and fluoranthene onto m-rGO@CS. Visibly, increasing the initial solution concentration of both adsorbates led to higher adsorbed amounts; however, the magnitude of the change among C_o_ was different for each compound. For instance, a significant alteration in q_t_ was observed for each concentration in the FLT adsorption. In comparison, there was no substantial change in adsorption capacity for anthracene between the two lowest concentrations.

The adsorption kinetic curves of anthracene and fluoranthene show a rapid increase at the beginning of the process, followed by a steady rise until equilibrium. The uptake of FLT by m-rGO@CS exhibited a longer and more stable growth throughout the process and was slightly superior to that of ANT by the same adsorbent; however, it took almost twice as long to reach equilibrium (Supplementary Material). For the uptake of FLT, the equilibrium times were 690, 870, and 1320 min for Co = 2, 3.5, and 5 mg/L, respectively. In contrast, the equilibrium times for ANT were 300, 330, and 360 min for the respective initial concentrations of 2, 3.5, and 5 mg/L. The faster adsorption rate of ANT can be credited to its higher affinity, as reported by de Farias et al. (2024), and its smaller molecular size. In the assays conducted at the two highest concentrations, the FLT was more efficiently adsorbed by the green beads (also on a molar basis). Besides its lower molecular mass, this result may be related to the higher hydrophobicity and stronger π-π interactions of this compound with the adsorbent (Adeola and Forbes 2019). Thus, it is plausible to assume that the sorption of PAHs onto m-rGO@CS is a multifaceted process involving pore filling, π–π stacking between the aromatic rings of the PAHs and the conjugated domains of rGO, together with hydrophobic partitioning effects (Cheng et al. 2019). The slight shifts observed in the FTIR and XPS spectra (Fig. 3b, FTIR, and Fig. 4, XPS) after adsorption support the occurrence of such non-covalent interactions. (Zhou et al. 2019). This interpretation is further supported by the thermodynamic and isosteric heat results, which suggest spontaneous and exothermic adsorption with magnitudes consistent with physical interactions dominated by π–π stacking and hydrophobic forces.

Table 2 displays the parameters of the kinetic models adjusted to the experimental data. Comparing the chemical reaction-based models, namely pseudo-first-order (PFO) and pseudo-second-order (PSO) models, PSO best described the adsorption of both compounds, as can be noted by the higher adjusted R^2^ values. This result indicates that the adsorption rate is primarily governed by the availability of active sites and surface interactions rather than by the adsorbate concentration in the solution, suggesting a surface-controlled process consistent with π–π stacking and hydrophobic interactions between the PAH molecules and the aromatic domains of rGO. Studies addressing the adsorption of different PAHs onto graphene wool (Adeola and Forbes 2019), activated carbons (Alves et al. 2022) and porous carbon (Cheng et al. 2019) also reported that the PSO order model has the finest fit to describe the experimental data.

**Table 2:** Kinetic parameters of anthracene and fluoranthene adsorption onto m-rGO@CS

Model	Parameters	ANT			FLT		
		2 mg/L	3.5 mg/L	5 mg/L	2 mg/L	3.5 mg/L	5 mg/L
	q_e_ (mg/g)	3.702	4.639	8.316	3.312	6.559	9.219
PFO	k_1_ (min^−1^)	0.103	0.146	0.078	0.182	0.005	0.007
	q_e_ (mg/g)	3.822	4.544	7.306	2.889	6.096	9.121
	R^2^ (-)	0.906	0.817	0.882	0.765	0.964	0.993
PSO	k_2_ (g.mg^−1^.min^−1^)	0.184	0.223	0.101	0.259	0.005	0.009
	q_e_ (mg/g)	3.983	4.759	7.862	2.992	7.379	10.317
	R^2^ (-)	0.907	0.882	0.937	0.856	0.982	0.990
Boyd’s	B (-)	0.184	0.104	0.268	0.156	0.127	0.180
	D_i_ (m^2^. min^−1^)	8.10E-9	4.57E-9	1.18E-8	6.87E-9	5.59E-9	7.94E-9
	R^2^ (-)	0.485	0.778	0.917	0.898	0.856	0.931
IPD	k_i_ (mg.g^−1^.min^−0.5^)	0.646	0.173	0.188	0.049	0.185	0.288
	C (mg/g)	0.289	2.869	4.528	2.008	1.11	2.527
	R^2^ (-)	0.879	0.972	0.893	0.978	0.983	0.938
EMTR	k_FM_ (m.min^−1^)	0.105	0.112	0.057	0.131	0.004	0.006
	R^2^ (-)	0.962	0.968	0.945	0.886	0.988	0.997
LDF	k_h_ (min^-1^)	0.045	0.045	0.050	0.0015	0.0013	0.003
	D_ldf_ (m^2^. min^−1^)	1.2E-9	1.2E-9	1.3E-9	3.9E-11	3.45E-11	7.8E-11
	R^2^ (-)	0.956	0.869	0.853	0.796	0.921	0.967
FD	D_fd_ (m^2^.min^-1^)	7.5E-10	1.95E-10	4.2E-10	3.9E-12	1.2E-11	4.5E-11
	R^2^ (-)	0.964	0.845	0.952	0.91	0.979	0.989

**Fig. 7:**
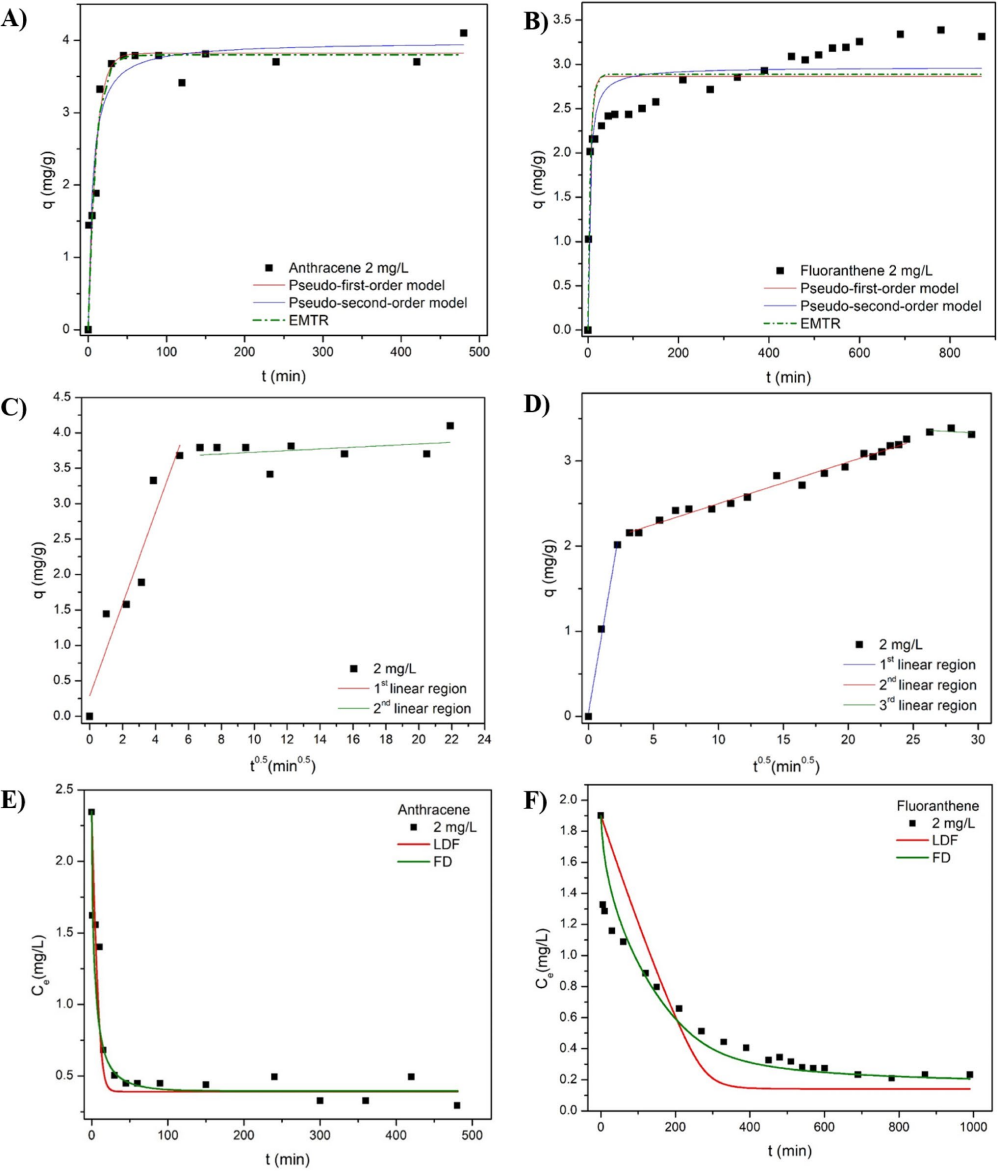
Adjustment of Pseudo-first order, Pseudo-second order, resistance to external mass transfer (EMTR), Intraparticle diffusion (IPD), Linear driving force (LDF), Fickian diffusion (FD) and Intraparticle diffusion models to the (**A**, **C** and **E**) anthracene and (**B**, **D** and **F**) fluoranthene adsorption. (C_o_ = 2 mg/L; 25 °C)

Besides the PFO and PSO models, phenomenological approaches were also applied to gain further insights regarding the adsorption kinetics and possible adsorption mechanisms. Thus, transfer-based models were also fitted to the experimental data. All kinetic adsorption curves showed a pattern of a rapid decrease in solution concentration at the beginning of the process, followed by a slower decrease and then the equilibrium phase. Therefore, the adsorption rate decreased with the reduction in the driving force, which agrees with the linear driving force (LDF) model (Peel and Benedek 1981). In this study, the LDF and Fickian diffusion (FD) models were fitted to the experimental data (Fig. 7). The FD model, derived from a mass balance in a control volume of the adsorbent particle, best described the anthracene and fluoranthene uptake by the m-rGO@CS.

The influence of intraparticle diffusion (IPD) was evaluated using the model proposed by Weber and Morris (1963) based on Fick’s law of diffusion. The q_t_ versus t^0.5^ plots were multi-linear for all compounds at all concentrations, suggesting that the adsorption process is complex and comprises multiple steps (Zhu et al. 2016). Except for the ANT adsorption on the lowest initial C_o_ (Fig. 7c), all kinetic curves exhibited three stages: (i) surface diffusion, (ii) intraparticle diffusion and (iii) equilibrium. From the IPD model plots, it can be inferred that the adsorption of ANT and FLT onto the reduced graphene oxide/chitosan-based beads is not governed solely by intraparticle diffusion since the first linear region does not pass through the origin. The unsatisfactory adjusted R^2^ values also show that although IPD is an important adsorption step, it is not the primary controlling step (de Andrade et al. 2020). Fitting the Boyd diffusion model to the experimental data corroborates this trend. Since the straight line in the B_t_ versus t plots (see Supplementary material) does not pass through the origin, the intraparticle diffusion is not the only controlling mechanism; therefore, the adsorption is likely governed by film and IPD diffusions (Yao and Chen 2017). Thus, the fit of these phenomenological models suggests the ANT and FLT adsorption onto the bead has a hybrid control mechanism, involving both external diffusion and surface reaction. The first stage is associated with external mass transfer across the boundary layer, while the second corresponds to gradual intraparticle diffusion through the crosslinked structure of the chitosan and rGO matrix (de Andrade et al. 2020).

The Boyd model was also used to estimate the effective diffusivity (D_f_) of the adsorption processes via the slope in the B_t_ versus t plots. Herein, the LDF and FD models were also used to estimate D_f._ (Table S6 of the Supplementary Material) displays the effective diffusion determined by the Boyd, LDF and FD diffusion models. For fluoranthene adsorption, the magnitude of the D_f_ values is quite different among them, whilst for anthracene adsorption, the values are closer. Previous pieces of literature stated D_f_ values closer to those determined through LDF and FD models. For instance, Valderrama et al. (2008) reported that the effective diffusion coefficients of ANT and FLT on activated carbon are in the order of 10^−12^ m^2^/min. Similarly, Suzuki and Kawazoe (1975) described that the D_f_ of ANT on activated carbon is in the order of 10^−11^ m^2^/min. Overall, it is noticeable that the D_f_ values for ANT uptake are greater than those for FLT, which is consistent with the experimental data, given that the anthracene adsorption was faster.

From Table 2, it can be observed that the K_FM_ of the beads exhibits higher values for the lower initial concentration of both ANT and FLT, which can be attributed to the lower concentration gradient. The good fit of the EMTR model indicates that the resistance to external mass diffusion is relevant in the PAHs adsorption process. Optimizing the agitation rate can reduce the impact of this step since higher agitation may diminish the film thickness and ease the adsorbate transfer through the film.

These results indicate that the adsorption of ANT and FLT onto m-rGO@CS is primarily surface-controlled, with contributions from internal diffusion resistance. This mechanistic understanding is important from a practical perspective, as it suggests that optimizing bead dispersion, surface accessibility, and hydrodynamic conditions could significantly enhance adsorption rates in continuous or large-scale systems.

### Adsorption equilibrium

**Fig. 8:**
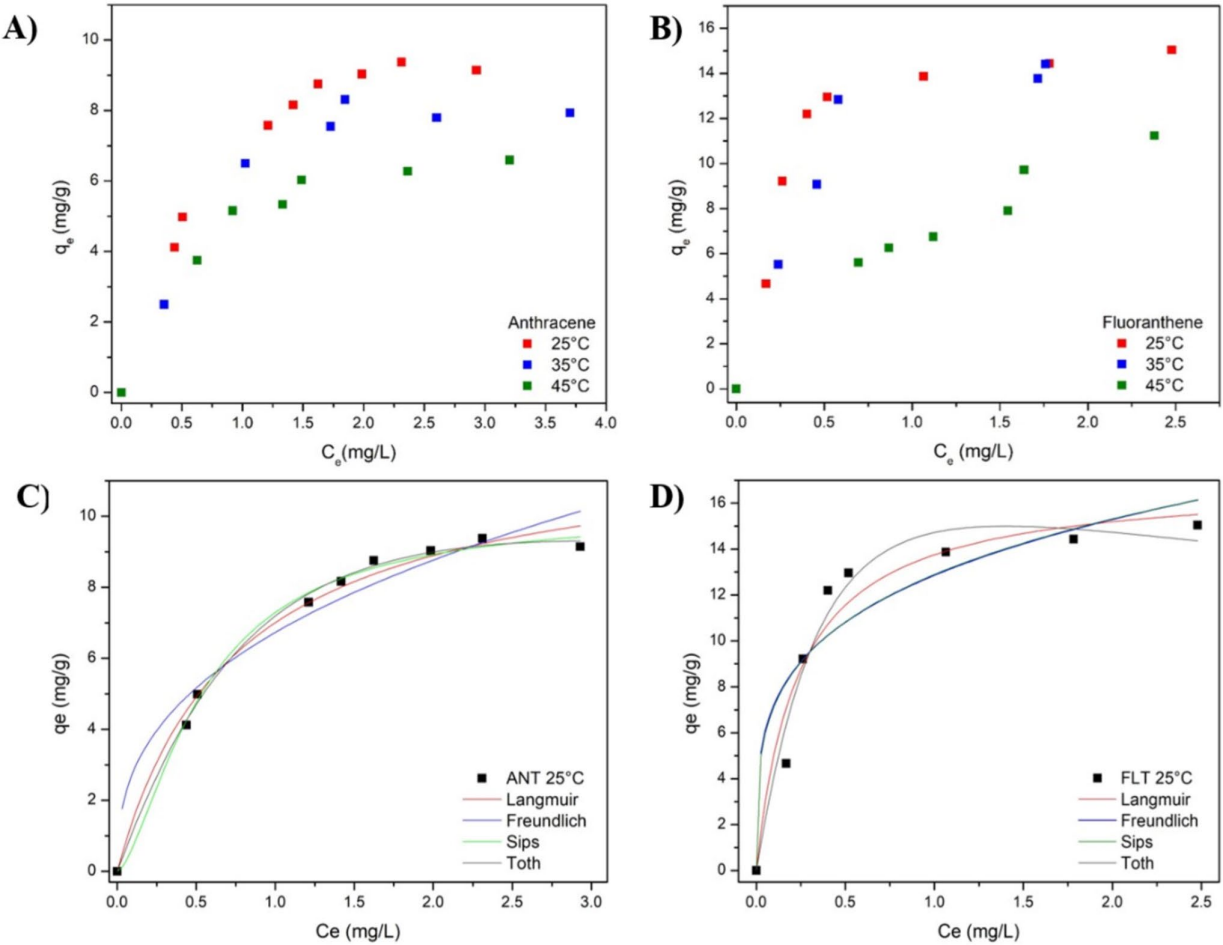
Equilibrium isotherms of (**A**) anthracene and (**B**) fluoranthene adsorption onto m-rGO@CS and isotherm model adjustments (**C**–**D**) to the 25 °C isotherm

Figure 8 displays the isotherms for anthracene and fluoranthene adsorption on m-rGO@CS at 25 °C, 35 °C and 45 °C. The assays were conducted for 24 h to ensure that equilibrium was reached. Perceptibly, the ANT and FLT adsorption isotherms have similar shapes and characteristics of a favourable process. According to the Giles (1960) classification, the ANT and FLT isotherms exhibit an L curve pattern, which indicates that there is no significant competition between the solvent and the adsorbate to occupy the available active sites of the adsorbent (Giles et al. 1960).

Notably, the adsorption processes of anthracene and fluoranthene were unfavoured by increasing temperature, indicating the exothermic nature of the process. Table 3 exhibits the parameters for the Langmuir, Freundlich, Sips, and Toth isothermal models, which were adjusted to the experimental data.

**Table 3:** Isotherm parameters of anthracene and fluoranthene adsorption onto m-rGO@CS beads

Model	Parameters	Anthracene			Fluoranthene		
		25 °C	35 °C	45 °C	25 °C	35 °C	45 °C
Langmuir	q_m_ (mg/g)	12.188	10.265	7.933	16.973	17.606	20.899
	K_L_ (L/mg)	1.34	1.41	1.697	4.265	2.631	0.471
	R^2^ (-)	0.989	0.954	0.985	0.940	0.944	0.974
Freundlich	K_F_ (mg/g)(L/mg)^1/n^	6.728	5.735	4.936	12.860	12.060	6.679
	n (-)	2.621	2.907	3.559	3.999	2.985	1.675
	R^2^ (-)	0.895	0.691	0.815	0.661	0.714	0.928
Sips	q_s_ (mg/g)	10.114	8.198	6.744	14.182	14.523	21.038
	K_s_ (L/mg)^n^	2.577	4.198	3.147	10.0	13.552	0.472
	n_s_ (-)	1.541	2.186	1.872	2.750	2.207	0.966
	R^2^ (-)	0.994	0.989	0.989	0.870	0.968	0.974
Toth	q_to_ (mg/g)	13.781	14.876	9.134	16.635	24.656	14.009
	b_t_ (L/mg)	1.589	2.989	1.309	0.478	3.786	1.296
	n_t_ (-)	1.351	1.664	1.274	1.307	3.389	0.139
	R^2^ (-)	0.996	0.966	0.986	0.959	0.982	0.912

The Sips and Toth isothermal models had a better correlation with the anthracene and fluoranthene adsorption data, as seen in Table 3, which is expected since they are three-parameter isotherms. The Sips model combines the Langmuir and the Freundlich isotherms and may indicate that the process occurs on an energetically heterogeneous surface. The heterogeneity of the systems is inferred by the *n*_*s*_ values that differ from unity (Shahri et al. 2018). The Toth model is a modification of the Langmuir model that has a greater accuracy between the experimental and predicted data due to accounting for the submonolayer coverage. It is widely applied to describe adsorption processes onto heterogeneous surfaces. Similarly to the Sips model, the n_t_ parameter of the Toth model deviating from unity indicates the heterogeneity of the adsorption system (Ayawei et al. 2017). Among the two-parameter isotherm models, the Langmuir model best described the experimental adsorption data of both contaminants, whereas the Sips was the best three-parameter isotherm.

Table 4 shows the maximum monolayer anthracene and fluoranthene adsorption capacity determined through the Langmuir isotherm model of various adsorbents. Evaluating the ANT adsorption, it is noticeable that m-rGO@CS exhibited comparable to or higher performance than that of other eco-friendly materials, such as biomass-derived biochar and marine Magnoliophyta (El Khames Saad et al. 2014; Ilyas et al. 2024); and a similar biopolymer/rGO-based material (Song et al. 2021b). As for fluoranthene adsorption, m-rGO@CS has a reasonable adsorption capacity compared with previous works with unconventional adsorbents. Compared to a similar graphene oxide/chitosan composite (Nascimento et al. 2024), m-GO@CS presents a slightly lower q_m_. Although certain adsorbents, such as activated carbons and modified graphene-based materials, exhibit higher maximum adsorption capacities, m-GO@CS can be considered a promising material and presents several advantages. Its synthesis employs *Eucalyptus* leaf extract as a green reducing agent, decreasing the use of toxic compounds. The green-synthesized material maintains good chemical stability, abundant oxygenated and amine functional groups from chitosan, and a uniform dispersion of magnetic particles that enable easy recovery from treated solution without centrifugation or filtration, which is necessary for other materials like the graphene oxide/chitosan suspension.

**Table 4:** Maximum anthracene and fluoranthene adsorption capacities of various adsorbents

Contaminant	Adsorbent	q_m_ (mg/g)	Reference
Anthracene	*Eucalyptus* derived biochar	0.141	(Ilyas et al. 2024)
	N-doped rGO/sodium alginate/polyvinylalcohol microbeads	1.43	(Song et al. 2021b)
	*Posidonia oceanica*	0.14	(El Khames Saad et al. 2014)
	Thermo-sensitive polymer based on magnetic GO	1.976	(Jamshidi et al. 2023)
	m-rGO@CS	12.19	This work
Fluoranthene	Magnetic chitosan/graphene oxide	28.22	(Nascimento et al. 2024)
	Lightweight expanded clay aggregate	0.007	(Asantewah et al. 2012)
	m-rGO@CS	16.97	This work

The composite also exhibited good regeneration performance after adsorption–desorption cycles, indicating its potential for practical application. Therefore, while the maximum adsorption capacity may not be the highest reported, the combination of green synthesis, facile separation, and reusability offers a favorable balance between efficiency, sustainability, and operational feasibility.

### Thermodynamic analysis

The estimation of the thermodynamic parameters, namely changes in Gibbs energy (ΔG), entropy (ΔS) and enthalpy (ΔH), is fundamental to understanding the physicochemical characteristics of the adsorption, as well as the spontaneity, favourability and randomness of the process. These parameters were calculated based on the data obtained from the equilibrium adsorption experiments conducted at 25 °C, 35 °C and 45 °C and displayed in Table 5. ΔS and ΔH were determined by the plot of ln Kc versus 1/T; the former was estimated from the slope of the graph, while the latter was calculated from the intercept (Queiroz et al. 2023). The negative ΔG values of the PAHs adsorption onto m-rGO@CS confirm the feasibility and spontaneity of the process. Furthermore, the increasing trend of ΔG values with the temperature rise indicates that higher temperatures do not favour the process (de Andrade et al. 2020). The negative ΔH values suggest that the adsorption of both ANT and FLT is exothermic, corroborating the results obtained in the equilibrium study (Fig. 8). Furthermore, their positive ΔS values show an increase in randomness, which can be attributed to the augmentation in the degrees of freedom of the adsorbed compounds at the solution/adsorbent interface, suggesting a structural reorganisation of the system during the adsorption process (Molina-Calderón et al. 2022). Besides the transition to a more disordered state, the rise in entropy may also suggest that physical interactions might be involved in the process (de Andrade et al. 2020). To further support the thermodynamic analysis, supplementary calculations using equilibrium constants derived from the Langmuir model were carried out (Table S7 of the Supplementary Material). The consistency between both approaches reinforces the reliability of the thermodynamic interpretation presented in this work.

**Table 5:** Thermodynamic parameters of anthracene and fluoranthene adsorption onto m-rGO@CS

Compound	Adsorbent	Temperature (K)	ΔH (kJ/mol)	ΔS (J/mol.K)	ΔG (kJ/mol)
Anthracene	m-rGO@CS	298	− 13.806	14.119	− 17.731
		308			− 18.154
		318			− 18.295
Fluoranthene	m-rGO@CS	298	− 14.770	21.701	− 20.803
		308			− 21.454
		318			− 21.671

These findings align with those of other studies in the literature that also evaluated the uptake of ANT and FLT. For instance, Banaeian and Mahdavian (2015), Ilyas et al. (2024) and Zango et al. (2019) evaluated the adsorption of anthracene onto carbon nanotubes, *Eucalyptus* wood biochar and metal–organic frameworks, respectively, and also determined that the process was exothermic and spontaneous. Similarly, Nascimento et al. (2024) and Akinpelu et al. (2019) corroborate that the uptake of fluoranthene by graphene oxide/chitosan composite and seagrass powder, respectively, was exothermic, feasible and spontaneous.

The isosteres of the ANT and FLT adsorption onto m-rGO@CS beads were determined at different fixed values of q_e_ (0.5, 1 and 1.5 mg/g) at 25 °C, 35 °C and 45 °C (Supplementary Material). The isosteric enthalpy of adsorption refers to the heat variation when an adsorbate binds to the adsorbent surface (Nuhnen and Janiak 2020). The values of ΔH_st_ for ANT and FLT adsorption (Table S8 S7) varied with increasing q_e_, suggesting that the active sites of the adsorbent surface are energetically heterogeneous, which agrees with the findings of the equilibrium study. As shown in Table S8 of the Supplementary Material, the ΔH_st_ values revealed a noticeable difference between anthracene (between –14.564 and –10.363 kJ/mol) and fluoranthene (between –77.880 and –75.438 kJ/mol). The lower magnitude for anthracene indicates weak, reversible physisorption dominated by π–π and van der Waals interactions, whereas the higher values for fluoranthene suggest stronger interactions, with possible formation of multilayers, due to its greater molecular size, polarizability, and hydrophobicity, which enhance π–π stacking and dispersion forces with the rGO domains. This behavior might justify the higher experimentally observed adsorption capacity of m-rGO@CS towards this FLT (Nuhnen and Janiak 2020).

### Simplified batch design

A simplified batch design was carried out to estimate the number of m-rGO@CS required to achieve 20–90% ANT and FLT reductions from different volumes of contaminant solutions (C_o_ = 10 mg/L). Fig. S22 displays the batch adsorption design for ANT and FLT uptake by the beads using a mass balance and the Sips equilibrium isotherm model, which best fits the experimental data.

As expected, the amount of adsorbent increases for both PAHs as the required removal efficiency increases. This pattern is more evident for ANT adsorption, as the amount of beads doubles when the required reduction changes from 60 to 90%, while the increase in adsorbent required for FLT adsorption shows a steady growth. Furthermore, the amount of adsorbent necessary for ANT uptake is about 1.8 times greater than that for FLT removal, corroborating the greater affinity between m-rGO@CS and FLT.

### Desorption and regeneration test

**Fig. 9:**
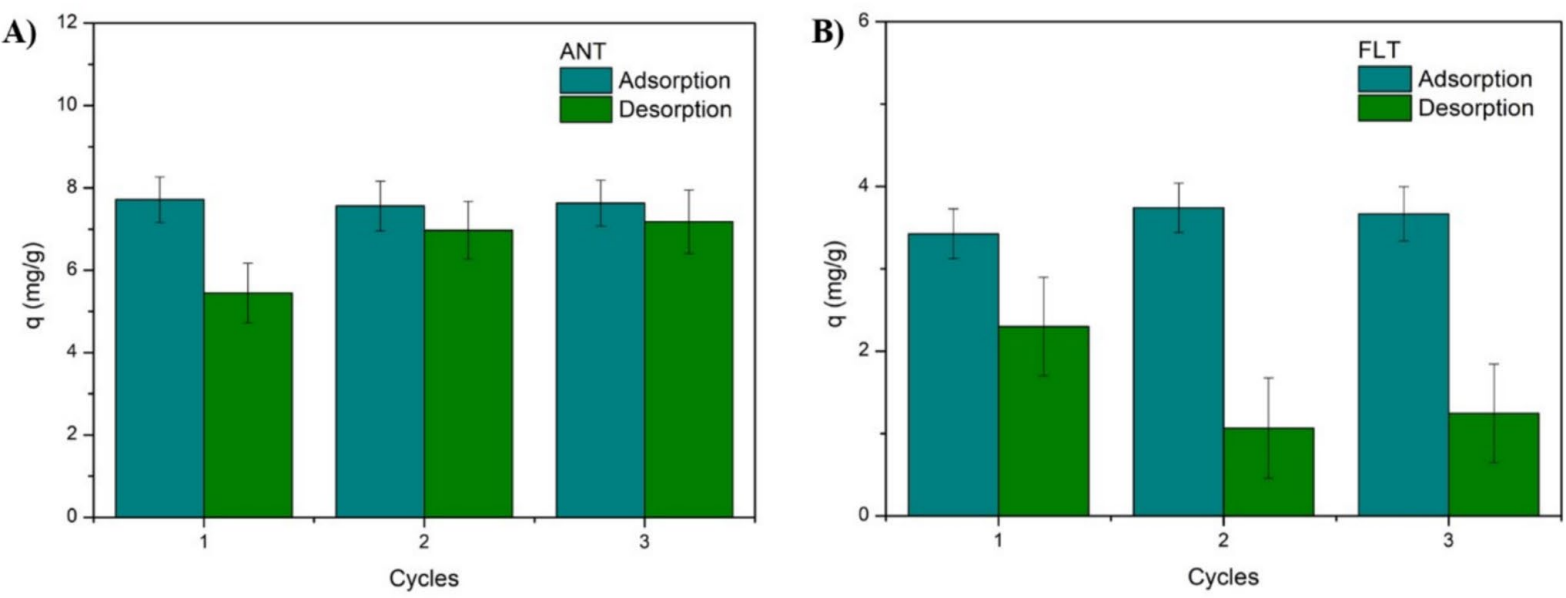
Adsorption and desorption capacities of (**A**) anthracene and (**B**) fluoranthene in three cycles

The capacity of the material to undergo regeneration is a fundamental attribute of an effective and practical adsorbent. The potential reuse of the same adsorbent in continuous cycles extends the lifetime of the material, reduces waste generation and enhances the cost-effectiveness of the process. PAHs are organic pollutants that can generally be dissolved in organic solvents due to their intermiscibility (Zhou et al. 2019). Ethanol was selected as the eluent for the regeneration studies due to its favorable environmental, safety, and economic characteristics compared with other commonly used solvents such as methanol and acetone. Among organic solvents, ethanol is considered a green solvent, as it can be obtained from renewable sources, is biodegradable, and exhibits low toxicity to humans and aquatic organisms. Furthermore, its intermediate polarity enables the effective desorption of hydrophobic organic molecules such as polycyclic aromatic hydrocarbons. Thus, in this work, ethanol was selected as the eluent for desorbing ANT and FLT without further optimization due to its balanced combination of efficiency, environmental sustainability, and cost-effectiveness, and based on its efficiency in previous studies, price and environmental aspects. Song et al. (2021a) and Queiroz et al. (2023) reported ethanol as a good eluent for removing anthracene and naphthalene, respectively, from graphene oxide-based materials. Figure 9 displays the adsorption and desorption cycles.

The adsorption capacities in both processes (anthracene and fluoranthene) remained similar throughout the three cycles, indicating a promising reusability capacity of the material. Conversely, the desorption performance was different for the two PAHs. The desorption capacity of ANT increased from the first to the second cycle and was kept constant. However, the desorption capacity of FLT decreased in each cycle, which might suggest a lower affinity between FLT and the eluent. Patiño-Ruiz et al. (2020), who also observed a low desorption capacity of PAHs from a chitosan-based composite, attributed this result to the stronger affinity between the compound and the bead’s surface. Additional investigations with other eluents may corroborate the regeneration ability of the material. Furthermore, the stability and reusability of the beads could be assessed in a continuous fixed-bed adsorption process, which is similar to that of industrial applications.

### Possible adsorption mechanisms

**Fig. 10:**
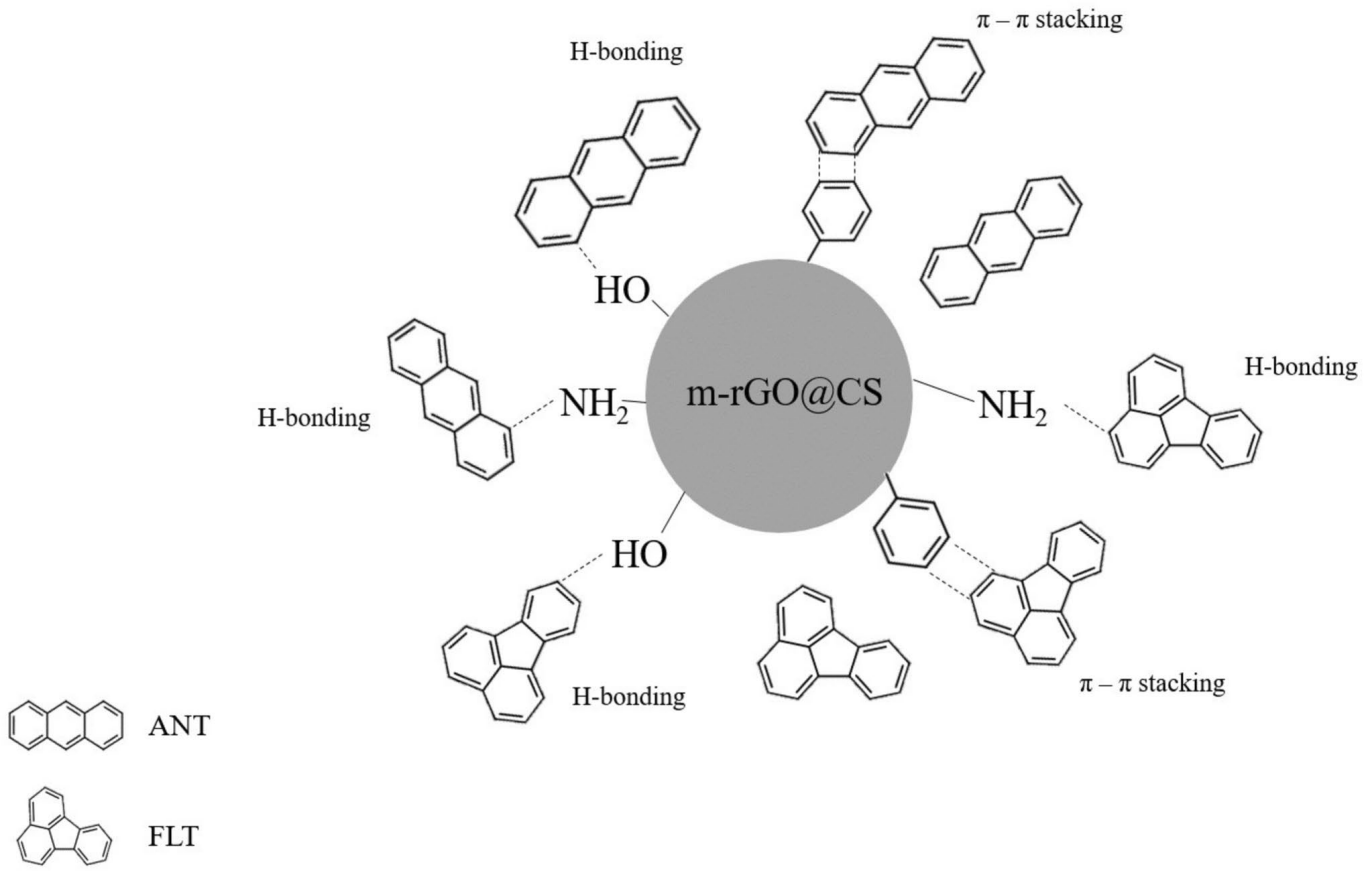
Schematic representation of possible adsorption mechanisms of anthracene and fluoranthene onto m-rGO@CS beads

The results presented herein indicate that the uptake of both contaminants by m-rGO@CS beads has multiple steps. Surface diffusion and intraparticle diffusion are most likely the primary controlling stages of the process. At first, ANT and FLT interact with the active sites of the adsorbent surface; then, in a slower phase, the contaminants diffuse into the material’s pores. These adsorbent-adsorbate interactions can occur through mechanisms of hydrogen bonding, π-π interactions and hydrophobic effects (Fig. 10), as suggested by the characterization analyses. Previous literature also indicates that polycyclic hydrocarbon adsorption onto carbon-based material happens through similar interactions. Prior research was reported by Queiroz et al. (2023), who evaluated the naphthalene adsorption on a magnetic chitosan/graphene oxide composite; Zhao et al. (2014), who used graphene to uptake phenanthrene; and Nascimento et al. (2024), who targeted fluoranthene adsorption onto a graphene oxide/chitosan composite. Furthermore, Patiño-Ruiz et al. (2020) and Rezagholizadeh-shirvan et al. (2024), who evaluated a chitosan-based bead, reported that the acceptor–donor type of interactions between chitosan functional groups and PAH contribute to the material's adsorption capacity.

## Conclusions

This paper evaluated the adsorption capacity of green-synthesized beads based on reduced graphene oxide, chitosan and iron (m-rGO@CS) for the uptake of anthracene (ANT) and fluoranthene (FLT). m-rGO@CS was efficiently used for the removal of ANT and FLT from low-concentration solutions. Experimental kinetic and equilibrium data were well described by pseudo-second order and Sips models. Phenomenological approaches to the kinetic data suggest that the adsorption of the contaminants onto the beads is a complex process that is likely governed by a combination of film and intraparticle diffusion mechanisms. The ANT and FLT adsorption are spontaneous, feasible and exothermic processes. Characterization analysis confirmed the adsorption of anthracene and fluoranthene on the beads and indicated that this occurred through hydrogen bonding and π-π interactions. Reusability tests showed that the green-synthesized adsorbent can be used for up to three cycles without a considerable difference in the adsorption capacity. Further assays on packed continuous columns are recommended to evaluate the stability and reusability of the materials.

## Supplementary Information

Below is the link to the electronic supplementary material.

ESM 1 (DOCX 4.66 MB)

## Data Availability

Not applicable.
