# Supplementary material for: Green-synthesized reduced graphene oxide@chitosan beads for the removal of polycyclic aromatic hydrocarbons
Source: Environ Sci Pollut Res Int. 2026 Jan 21;33(4):1332–52. doi: 10.1007/s11356-025-37364-6 (PMC12901273; doi:10.1007/s11356-025-37364-6)
Supplement: Supplementary file 1 — (DOCX 4.66 MB) [file 11356_2025_37364_MOESM1_ESM.docx]

**Supplementary Material**

Title: Green-synthesized reduced graphene oxide@chitosan beads for the removal of polycyclic aromatic hydrocarbons: a complete batch study

Journal: Environmental Science and Pollution Research

Marina Barbosa de Farias^a,b,c^, Albertina Gonçalves Rios^b,c^, Alexandre Filipe Porfírio Ferreira^b,c^, Patrícia Prediger^d^, Melissa Gurgel Adeodato Vieira^a*^

^a^ Universidade Estadual de Campinas, School of Chemical Engineering, Albert Einstein Av. 500, 13083-852 Campinas, São Paulo, Brazil.

^b^ Universidade do Porto, LSRE-LCM – Laboratory of Separation and Reaction Engineering - Laboratory of Catalysis and Materials, Faculty of Engineering, Rua Dr Roberto Frias s/n, 4200-465 Porto, Portugal

^c^ Universidade do Porto, ALiCE – Associate Laboratory in Chemical Engineering, Faculty of Engineering, Rua Dr Roberto Frias s/n, 4200-465 Porto, Portugal.

^d^ Universidade Estadual de Campinas, School of Technology, 13484-332 Limeira, São Paulo, Brazil.

*Corresponding author’s email: melissagav@feq.unicamp.br (Melissa Gurgel Adeodato Vieira).

**Table S1.** Levels at which the factors were evaluated to determine their influence on the adsorption capacity of the beads

| **Factors** | **Levels** | | |
| --- | --- | --- | --- |
|  | **−1** | **0** | **+1** |
| pH (x_1_) | 4 | 6 | 8 |
| Adsorbent dosage (g/L) (x_2_) | 0.5 | 1 | 1.5 |
| Initial solution concentration (mg/L) (x_3_) | 2 | 5 | 8 |

**Table S2**. Kinetic models.

| **Kinetic models** | **Equation** |  | **Reference** |
| --- | --- | --- | --- |
| Pseudo-first order (PFO) | $q \left( t \right)=q_{e}\left( 1-e^{-k_{1}t} \right)$ | (1) | (Lagergren 1898) |
| Pseudo-second order (PSO) | $q \left( t \right)=\frac{k_{2}q_{e}^{2}t}{1+ k_{2}q_{e}t}$ | (2) | (Ho and McKay 2000) |
| Boyd’s model | $F\left( t \right)= \frac{q_{t}}{q_{e}}$  $Bt=-0.4977-ln(1-F)$  $B=\frac{\pi^{2}D_{i}}{r^{2}}$ | (3)  (4)  (5) | (Boyd et al. 1947) |
| Intraparticle diffusion (IPD) | $q_{t}=k_{i}t^{1/2}+C$ | (6) | (Weber and Morris 1963) |
| External mass transfer resistance (EMTR) | $\frac{dC}{dt}=-k_{TM}(C_{t}-C_{p}(t))$ | (7) | (Puranik et al. 1999) |

𝑞_𝑒_ : concentration in the solid phase at equilibrium (mg/g); 𝑘_1_: first-order rate constant (min^-1^); 𝑘_2_: second-order rate constant (g/mg.min);𝐶𝑡 : concentration in the bulk solution (mg/L); 𝐶𝑠 : concentration at the external surface (mg/L); 𝐵𝑡: mathematical function of 𝐹 (-); 𝐵: time constant (min^-1^); 𝐷𝑖: internal diffusion coefficient (m^2^ /min); 𝑟: radius of the adsorbent particles (m); 𝑘𝑖 : intraparticle diffusion constant (mg/g.min^0.5^); 𝐶: parameter of intraparticle diffusion model (mg/g); k_TM_ is the external mass transfer resistance (min^-1^); C_p_ is the adsorbate concentration at the interface between the liquid and the adsorbent as a function of time (mg/L).

The linear driving force (LDF) model is based on a mass balance in a batch adsorption system (8), in which the initial condition is $t=0 \to C=C_{o}\to\bar{q}=0$. The LDF model can be described by Equation 9.

$V\frac{dC}{dt}+m\frac{d\bar{q}}{dt}=0$ (8)

$\frac{d\bar{q}}{dt}=k_{h}(q_{e}-\bar{q})$ (9)

Where $\bar{q}$ is the average adsorbate concentration on the particle; q_e_ is the adsorbed concentration at equilibrium at a given instant, and k_h_ is the intraparticle mass transfer coefficient. The effective homogeneous diffusivity (D_if_) can be estimated by Equation 10, in which R is the particle radius.

${Dif}_{LDF}=\frac{k_{h}R^{2}}{15}$ (10)

In the Fickian Diffusion (FD) model, the mass balance is performed in a control volume inside the particle. For this, the boundary conditions are $r=0 \to\frac{\partial q}{\partial r}=0;r=R \to q=q^{*}$ and the initial conditions are $t=0\to q\left( r \right)=0$. The FD is described by Equation 11.

$\frac{\partial q}{\partial t}=\frac{Dif}{r^{2}}\frac{\partial}{\partial r}\left[ r^{2}\left( \frac{\partial q}{\partial r} \right) \right]$ (11)

**Table S3.** Equilibrium isothermal models.

| **Equilibrium model** | **Equation** |  | **Reference** |
| --- | --- | --- | --- |
| Langmuir | $q_{e}=\frac{q_{m}k_{L}C_{e}}{1+ k_{L}C_{e}}$ | (12) | (Langmuir 1918) |
| Freundlich | $q_{e}=k_{F}C_{e}^{\frac{1}{n}}$ | (13) | (Freundlich 1926) |
| Sips | $q_{e}=\frac{q_{s}\left( K_{s}C_{e} \right)^{n}}{1+\left( K_{s}C_{e} \right)^{n}}$ | (14) | (Sips 1948) |
| Toth | $q_{e}=\frac{q_{to}C}{\left( b_{t}+C^{n_{t}} \right)^{\frac{1}{n_{t}}}}$ | (15) | (Toth 1971) |

q_e_: adsorbate concentration in equilibrium on the adsorbent surface (mg/g); q_m_: maximum adsorption capacity (mg/g); C_e_: equilibrium concentration of the adsorbate (mg/.L); k_L_ is the Langmuir equilibrium constant associated with the affinity of the sites (L.mmol^-1^); k_F_ is the Freundlich constant [(mg/g).(L/mg)^1/n^]; n is the empirical constant associated with the adsorption intensity (-); q_to_ is the maximum adsorption capacity (mg/g); n_t_ is a parameter related to the heterogeneity of the adsorbent; b_t_ is a parameter related to the affinity towards the adsorbate (L/mg).

- Full-factorial design

**Figure S1. A)** Pareto chart (absolute values) and B) Plot of predicted versus observed values of anthracene adsorption.

**A)**


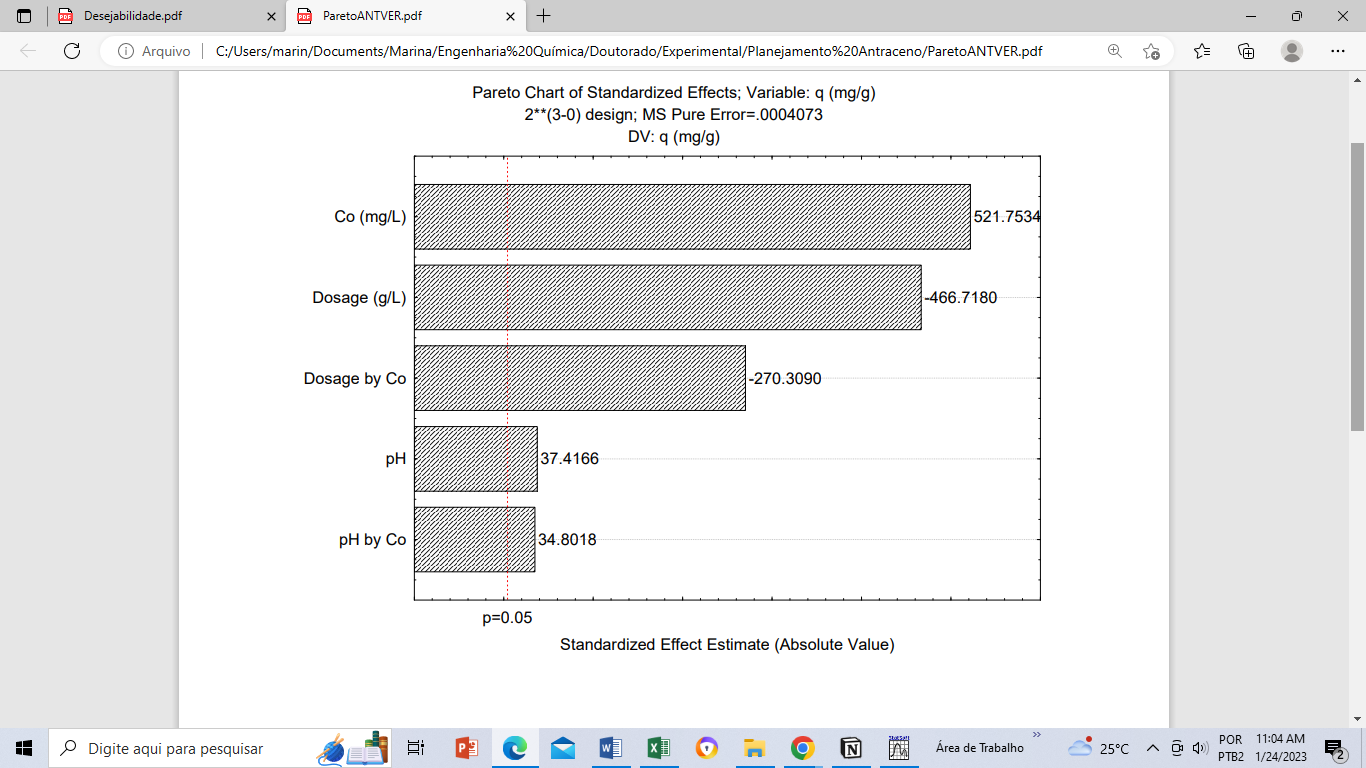

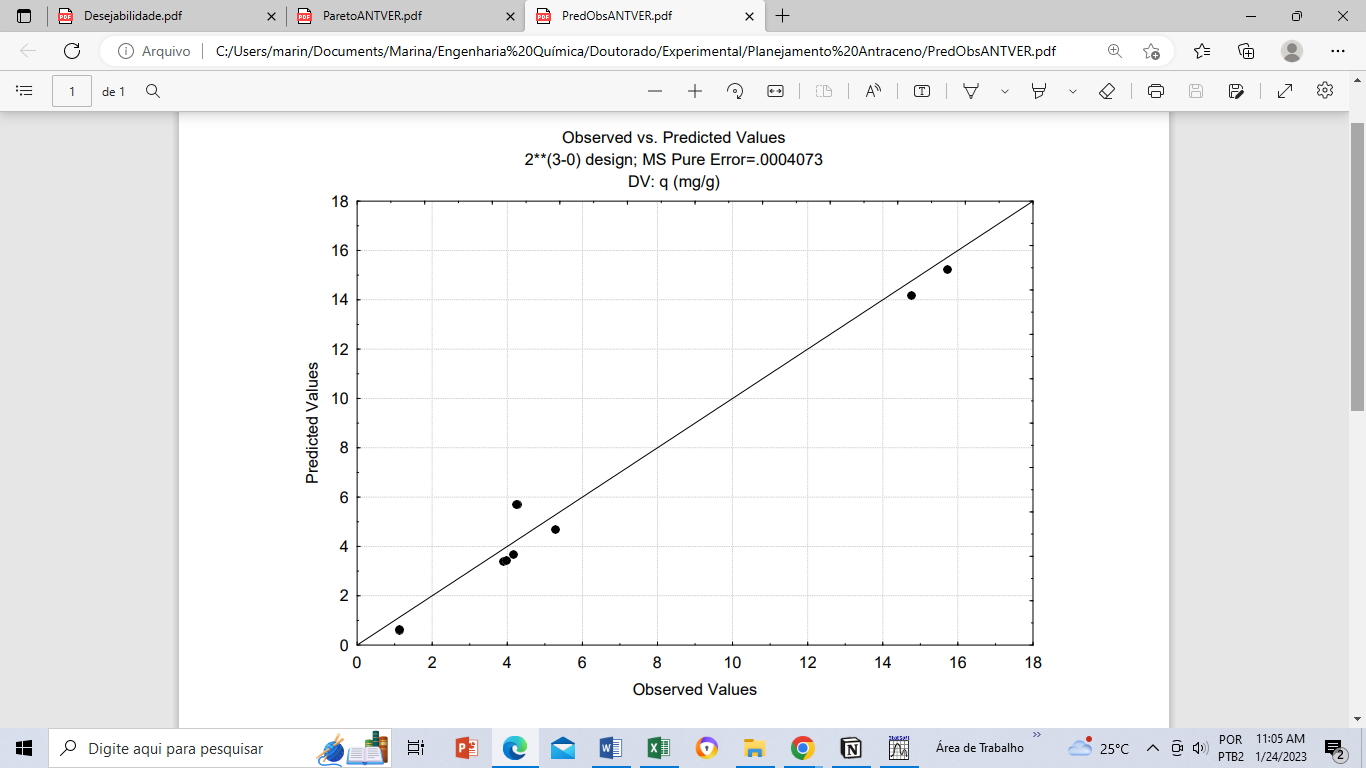


**B)**

**Figure S2.** Response surface plots of the interactions between a) Concentration and dosage; e b) pH and dosage (coded values) for anthracene adsorption.

**B)**

**A)**


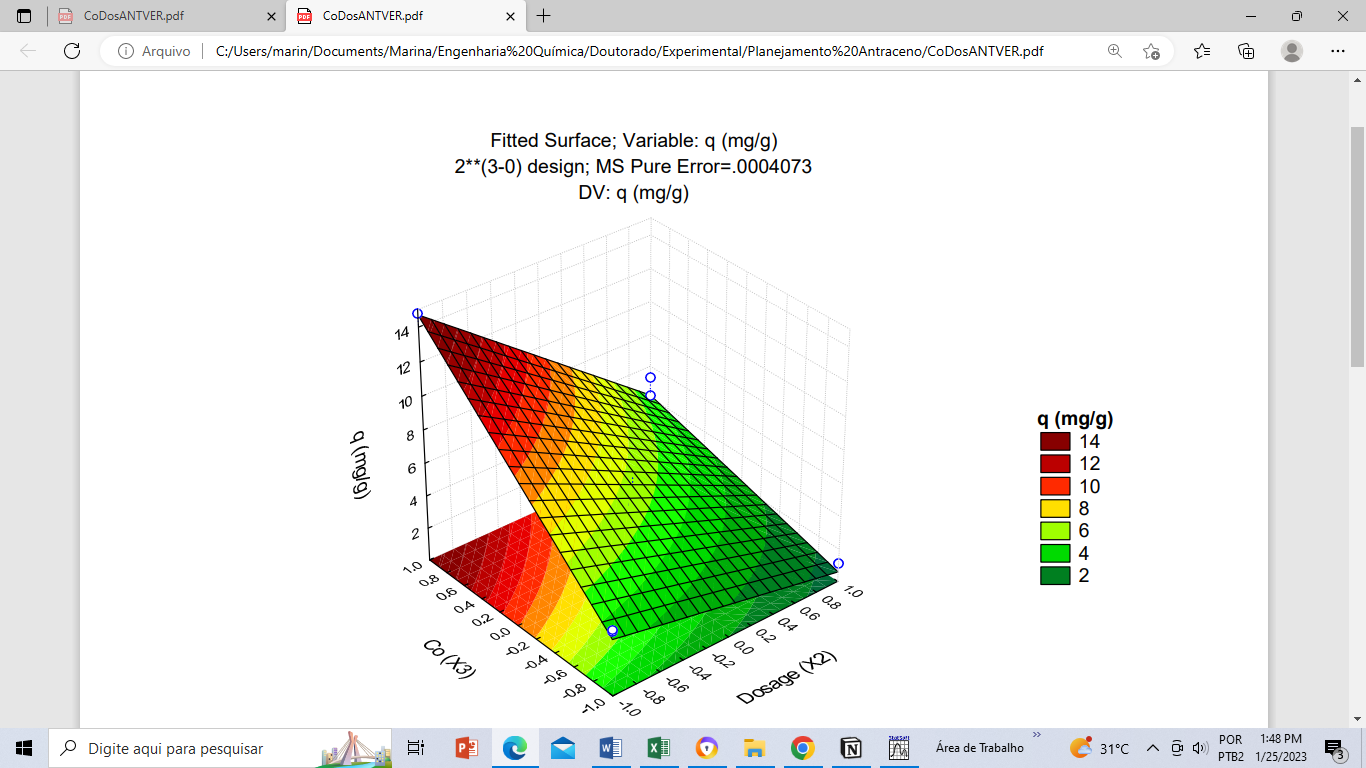

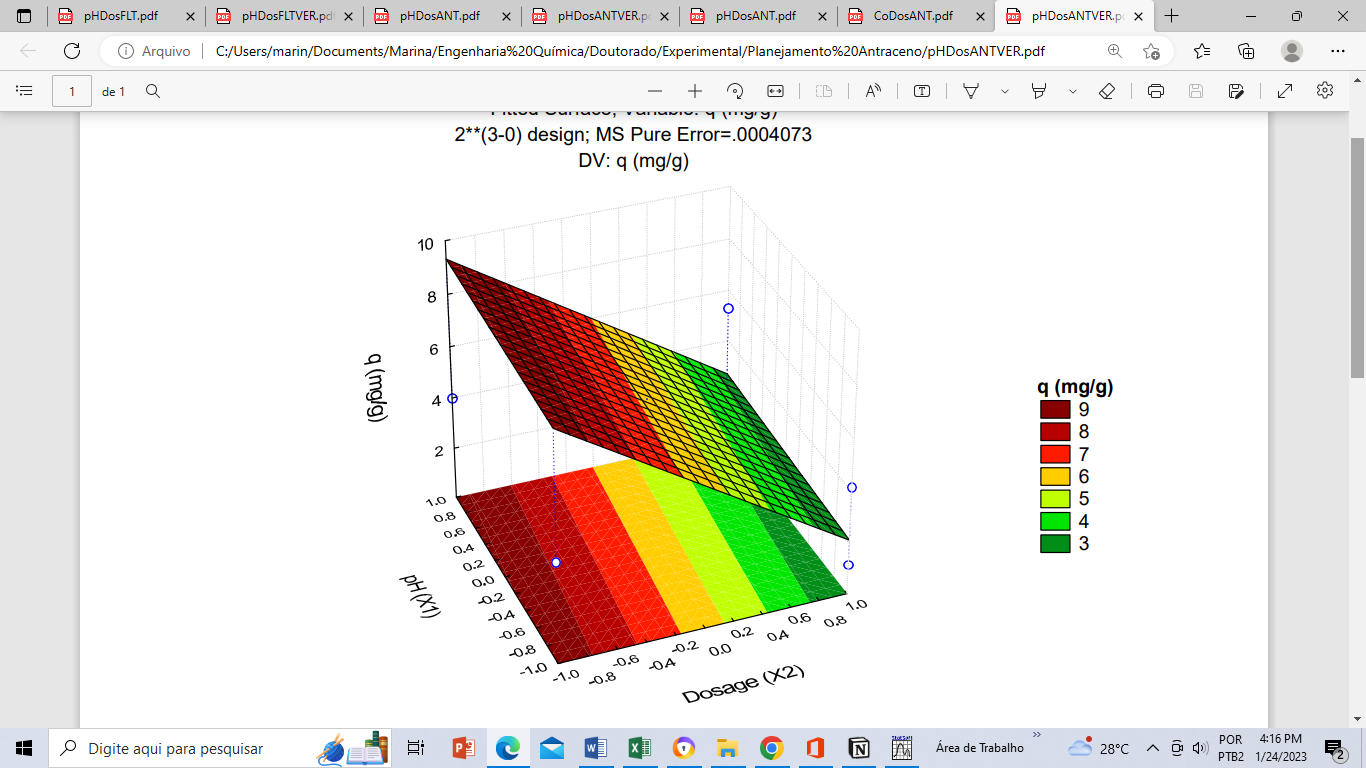


**Figure S3.** Profile of predicted values and desirability of anthracene adsorption.


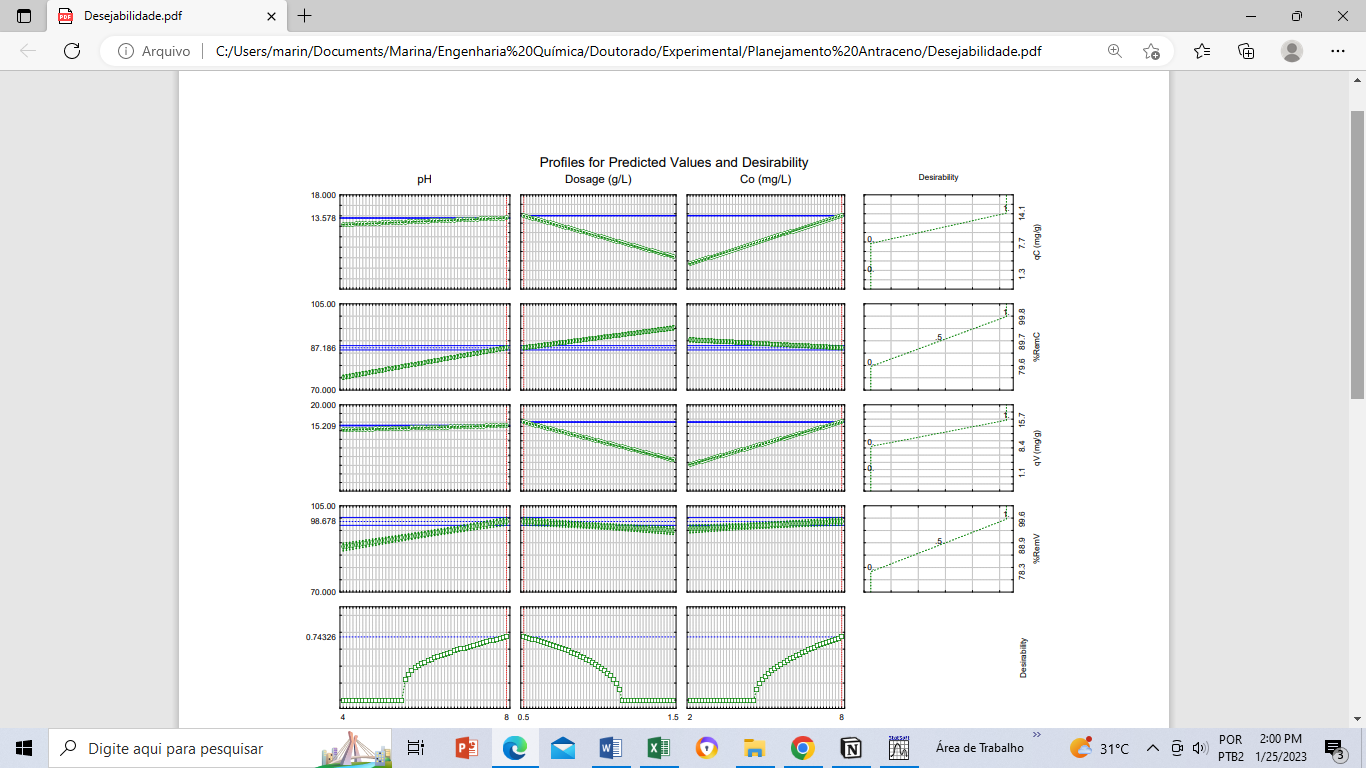


**Figure S4.** Pareto chart (absolute values) and B) Plot of predicted versus observed values of fluoranthene adsorption.

**B)**

**A)**


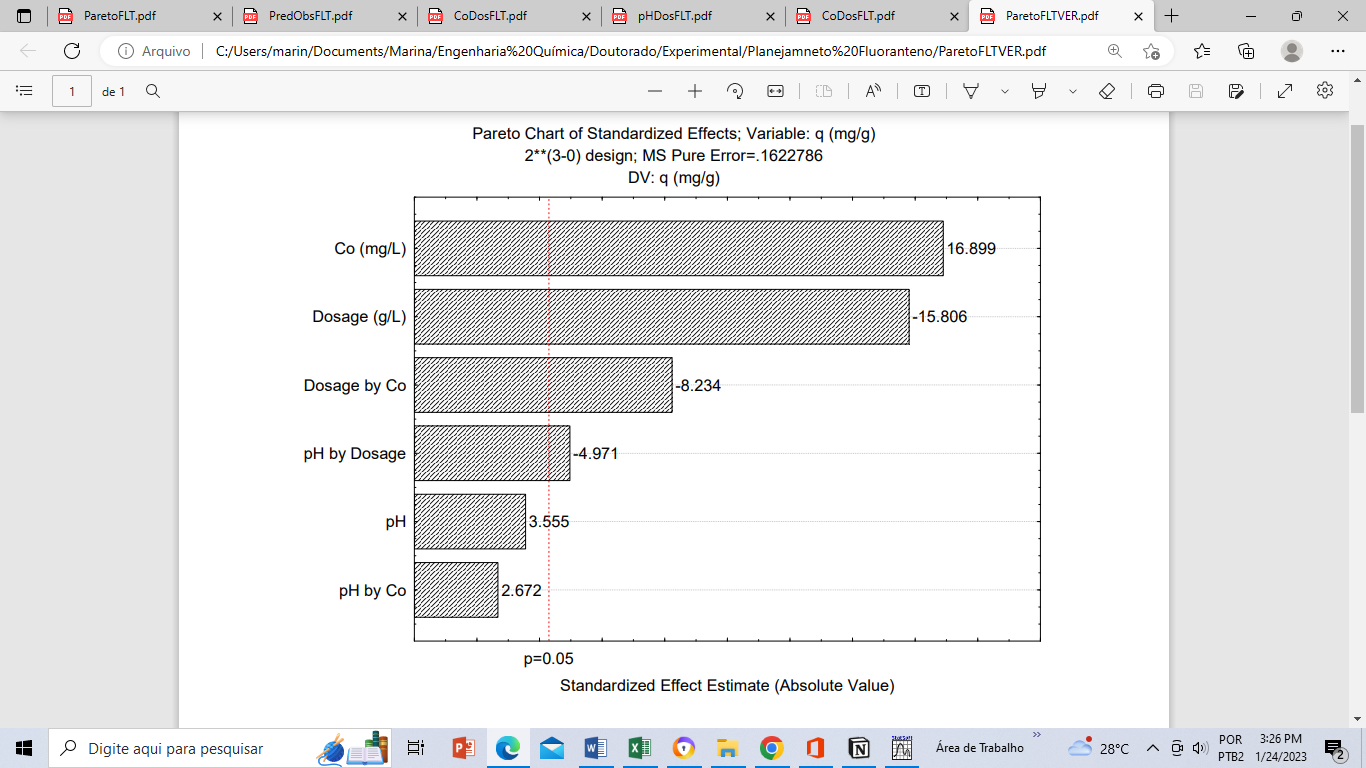

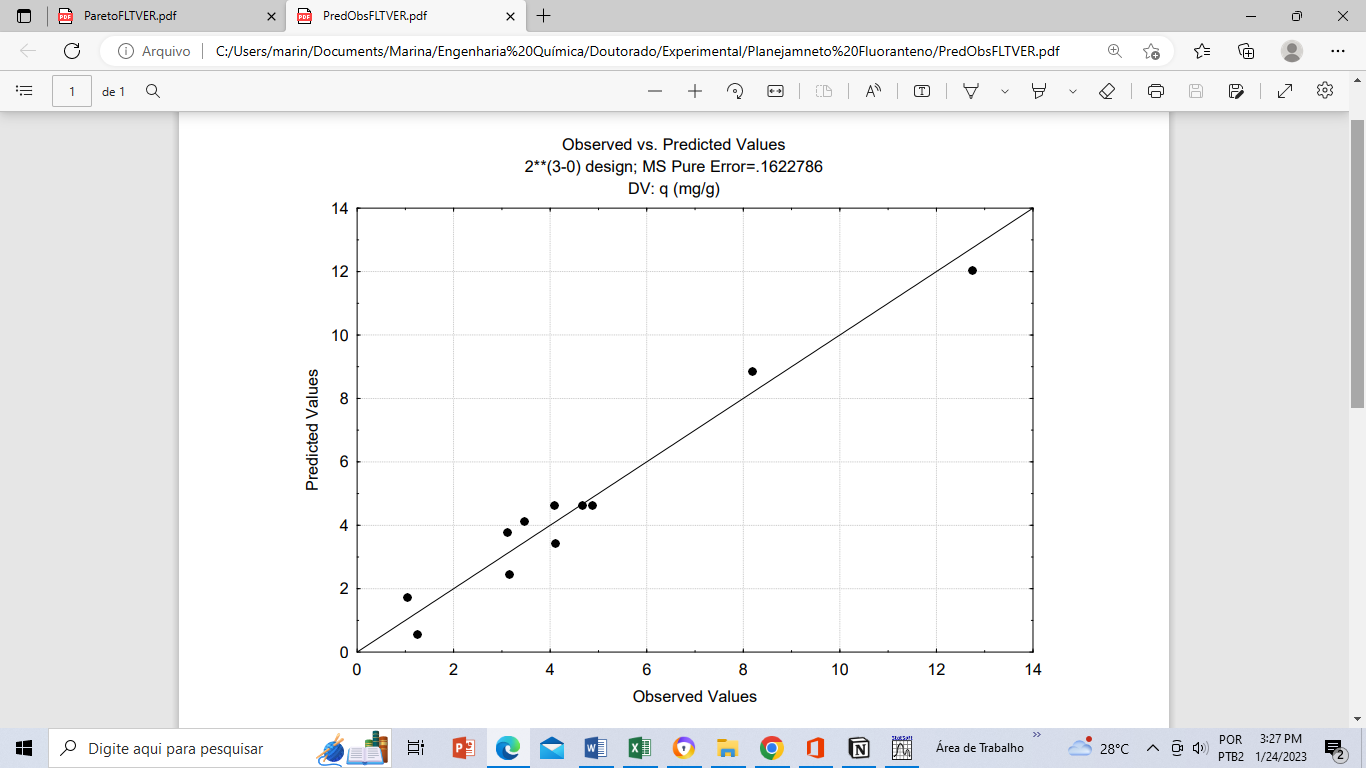


**Figure S5.** Response surface plots of the interactions between a) Concentration and dosage; e b) pH and dosage (coded values) for fluoranthene adsorption.


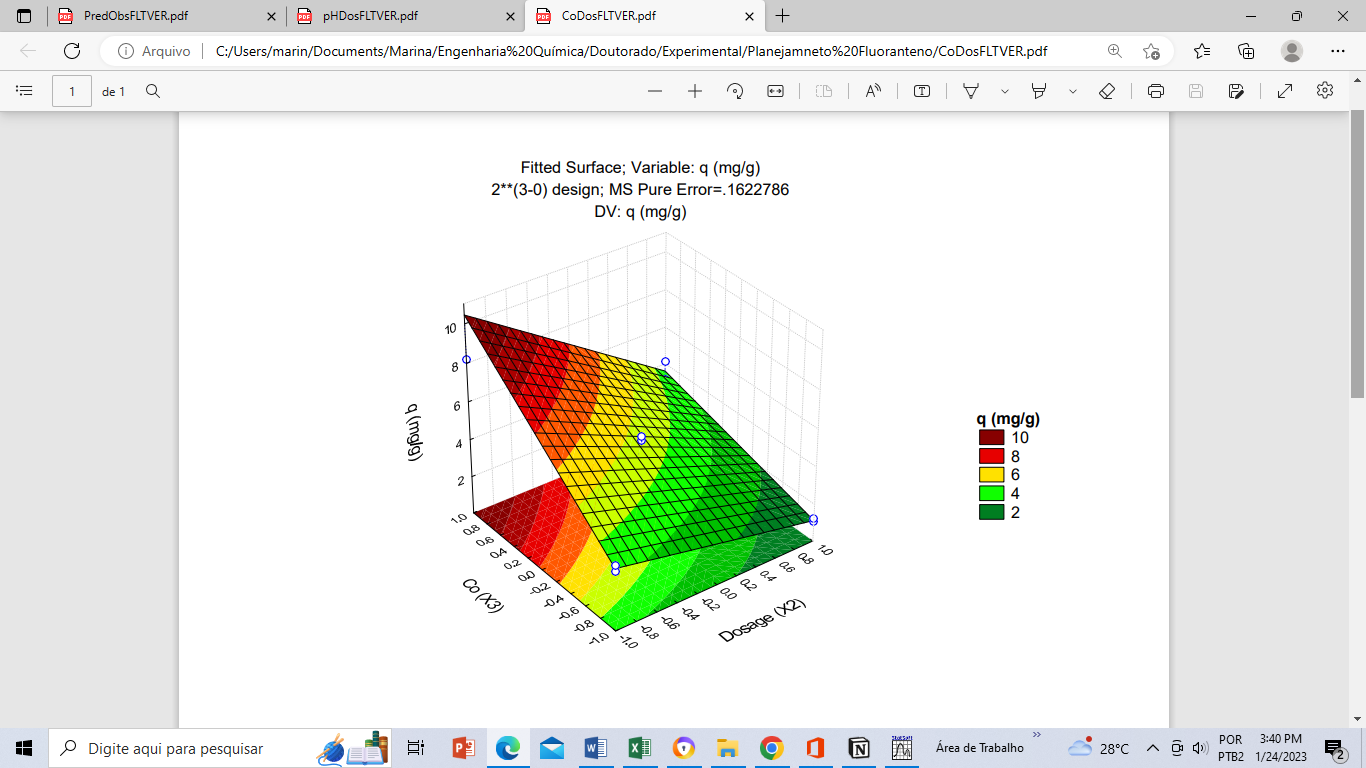

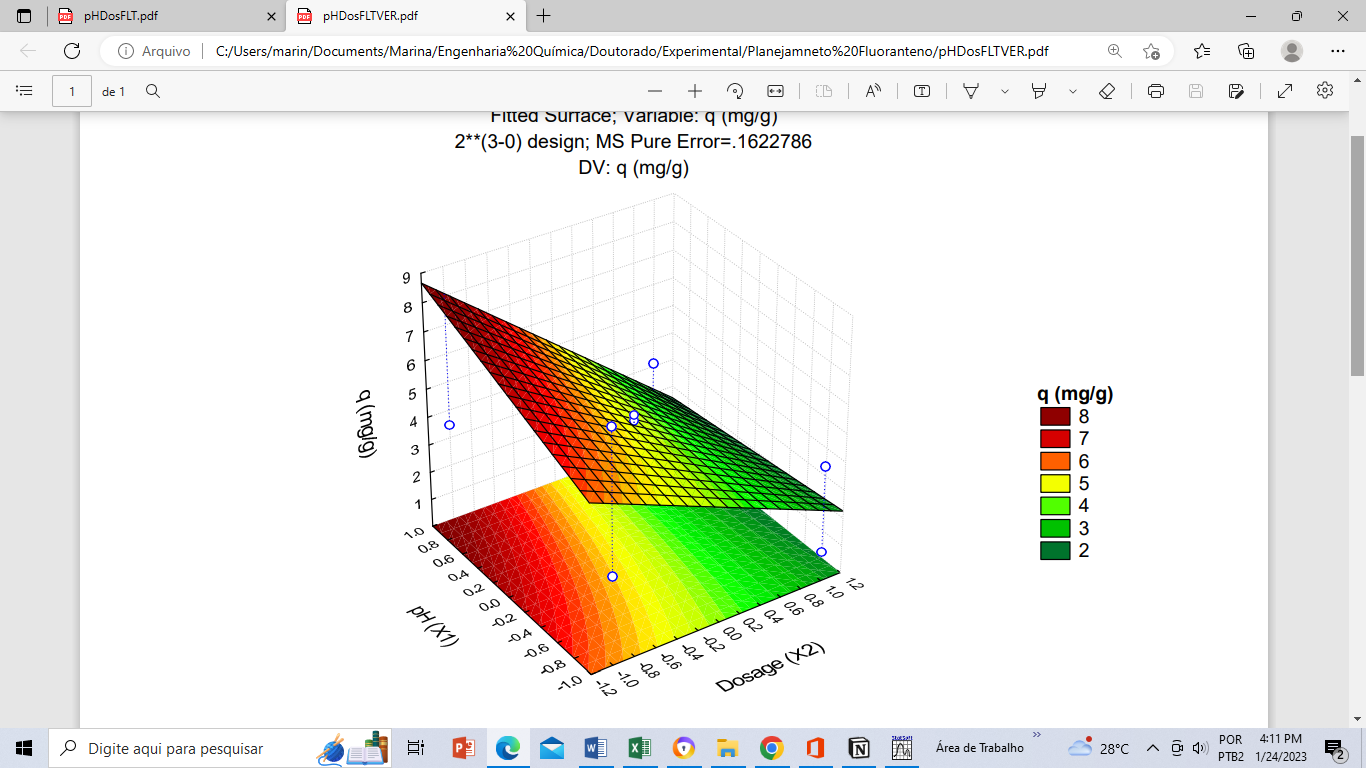


**A)**

**B)**

**Figure S6.** Profile of predicted values and desirability of fluoranthene adsorption.


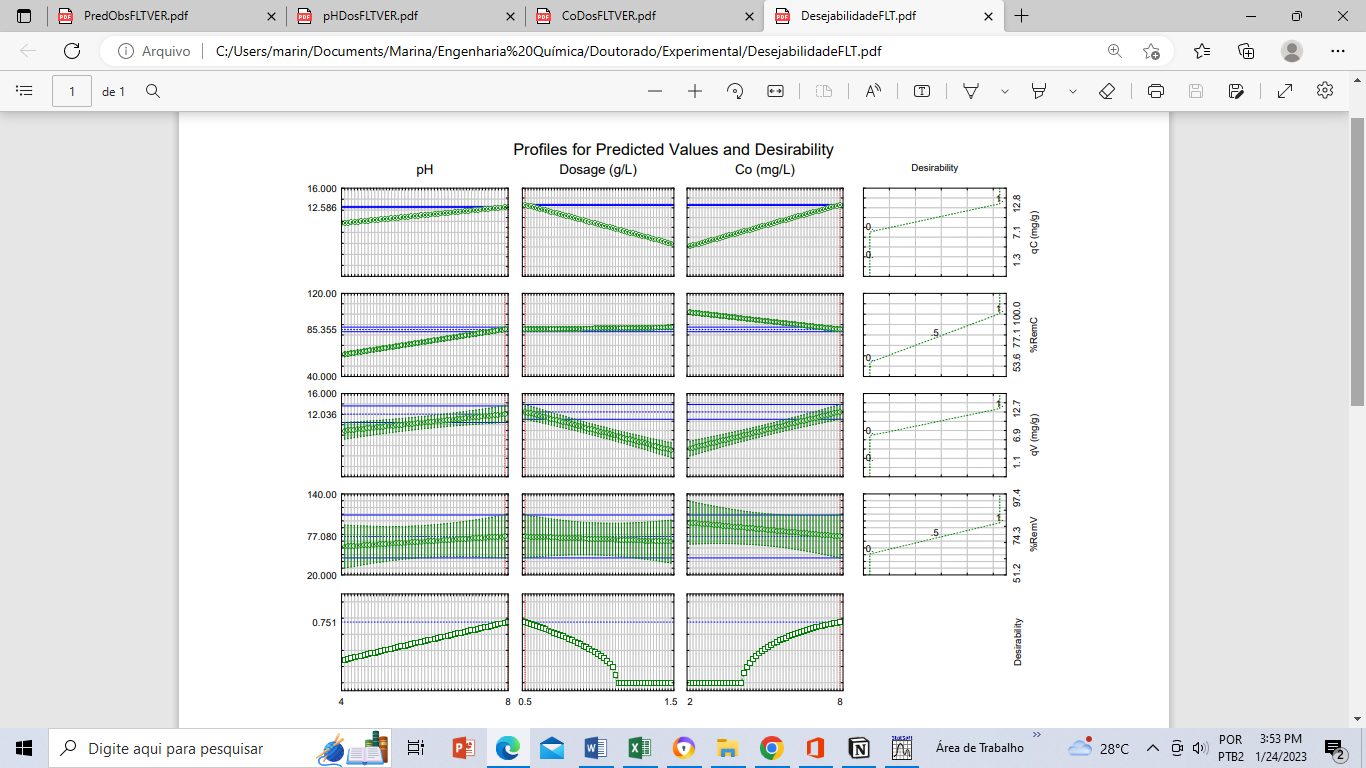


**Table S4.** ANOVA table of anthracene adsorption.

| **Source of variation** | **Sum of squares** | **Degrees of freedom** | **Mean squares** | **F_5,5_** | **F_5,5tabled_** |
| --- | --- | --- | --- | --- | --- |
| **Regression** | 230.4045 | 5 | 46.0809 | 26.55 | 5.05 |
| **Residues** | 8.6774 | 5 | 1.7355 |  |  |
| **Lack of fit** | 8.6766 | 3 | 2.8922 |  |  |
| **Pure error** | 0.0008 | 2 | 0.0004 |  |  |
| **Total** | 239.0818 | 10 |  | | |

**Table S5.** ANOVA table of fluoranthene adsorption.

| **Source of variation** | **Sum of squares** | **Degrees of freedom** | **Mean squares** | **F_5,5_** | **F_5,5tabled_** |
| --- | --- | --- | --- | --- | --- |
| **Regression** | 105.1064 | 6 | 17.5177 | 4.76 | 4.53 |
| **Residues** | 14.7079 | 4 | 3.6770 |  |  |
| **Lack of fit** | 3.7043 | 2 | 1.85216 |  |  |
| **Pure error** | 0.3246 | 2 | 0.16228 |  |  |
| **Total** | 109.1352 | 10 |  |  |  |

- **Kinetic adjustments**

**Figure S7.** Pseudo-first order, pseudo-second order and external mass resistance models fit for anthracene adsorption at A) 2 mg/L, B) 3.5 mg/L, and 5 mg/L.

**
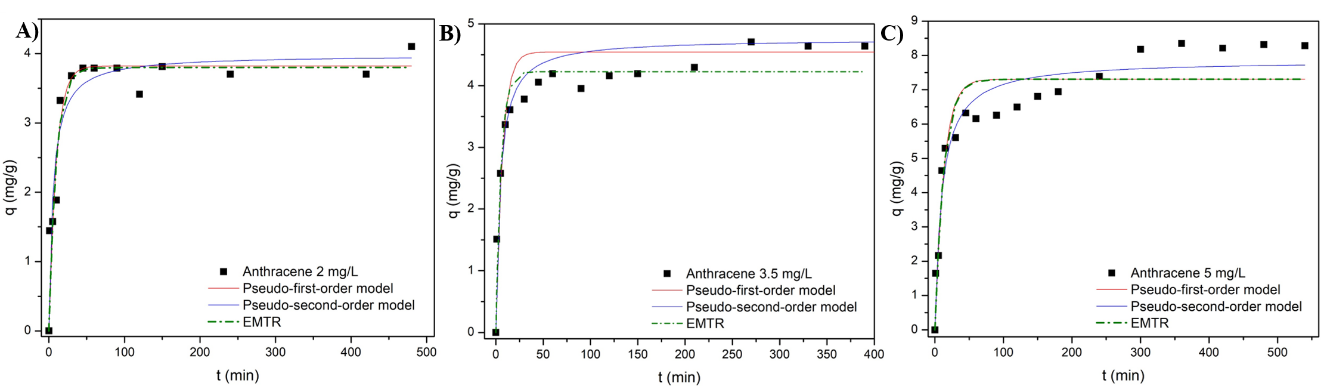
**

**Figure S8.** Pseudo-first order, pseudo-second order and external mass resistance models fit for fluoranthene adsorption at A) 2 mg/L, B) 3.5 mg/L, and 5 mg/L.

**
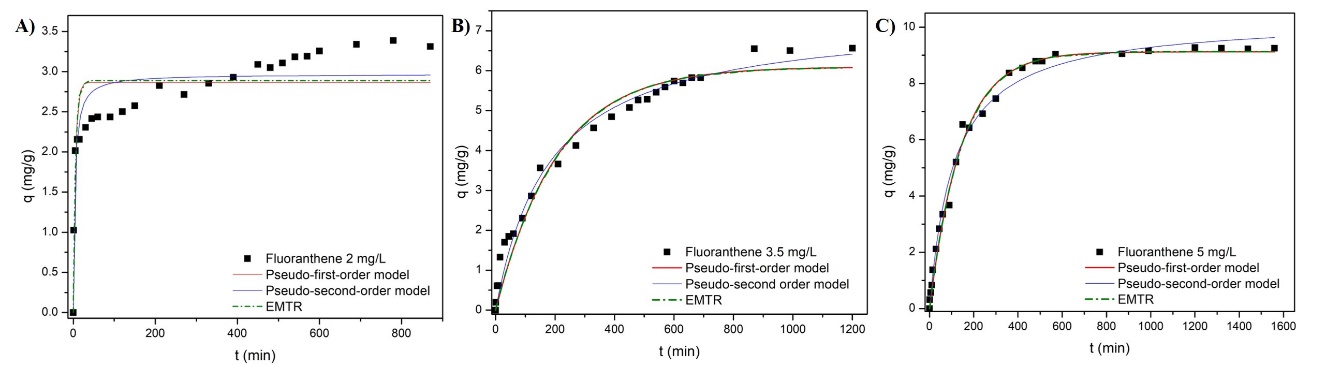
**

**Figure S9.** Adjustment of Boyd and intraparticle diffusion models for fluoranthene adsorption.

**
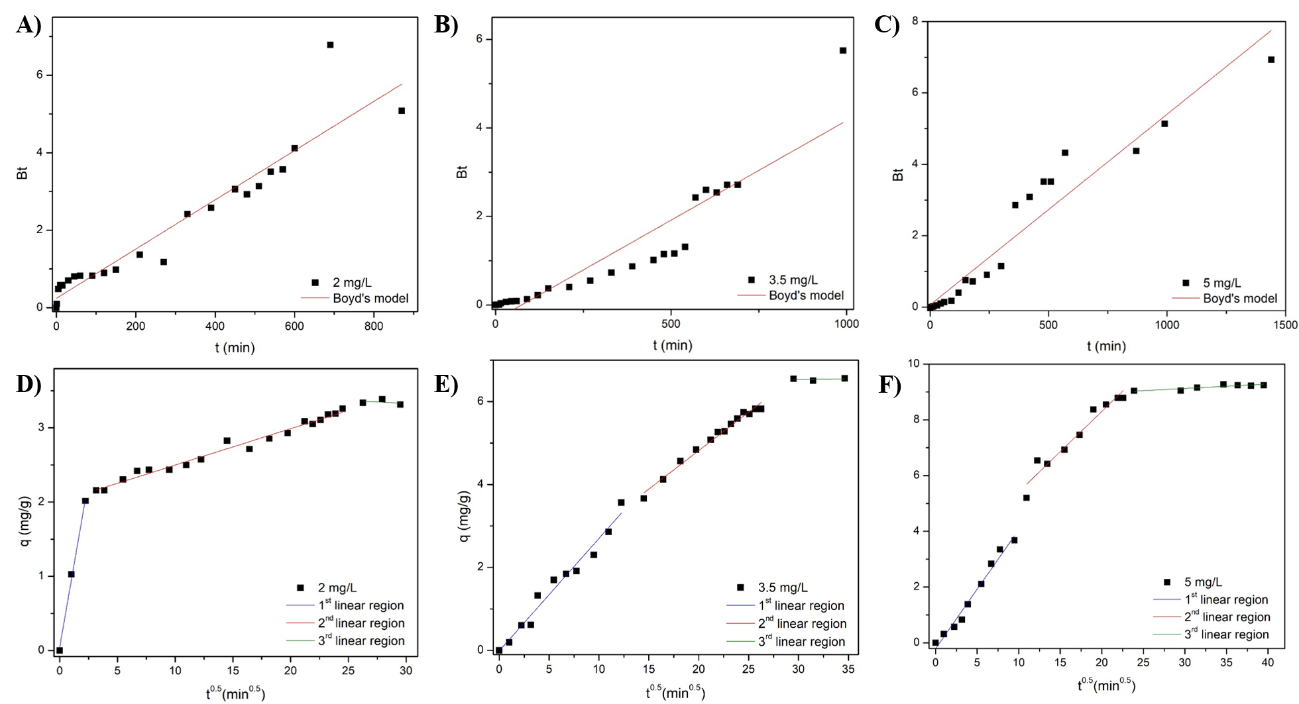
**

**Figure S10.** Adjustment of Linear driving force and Fickian diffusion models for A) anthracene and B) fluoranthene adsorption at 2 mg/L.

**
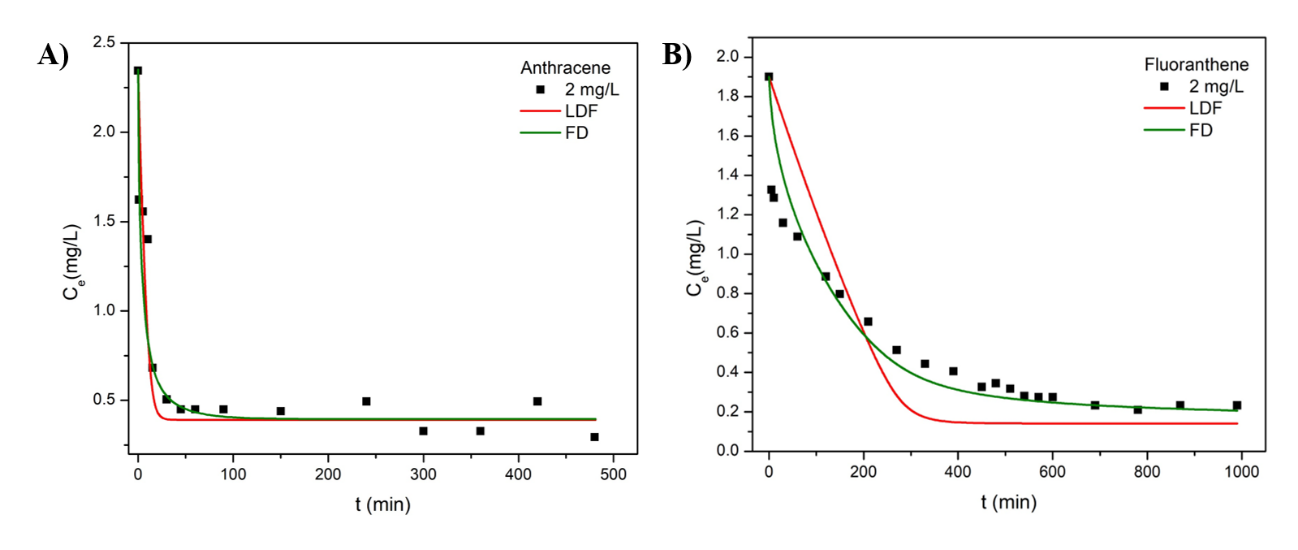
**

**Figure S11. Comparison of anthracene and fluoranthene relative adsorption values qt/qs vs. time at A) 2 mg/L, B) 3.5 mg/L and C) 5 mg/L (where qt is the amount adsorbed in a certain time and qs is the saturated amount).**

**
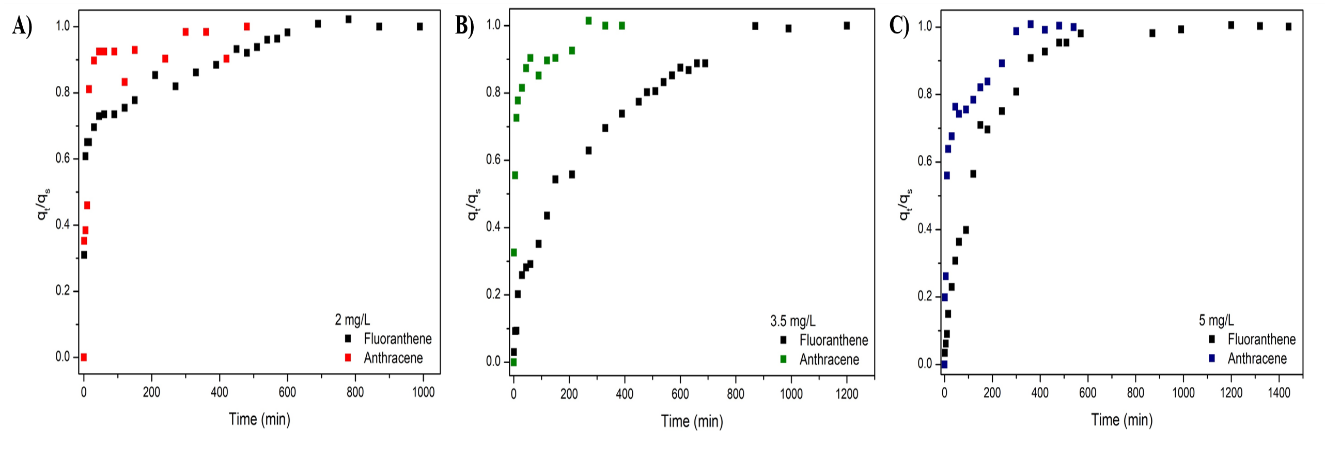
**

**Figure S12. Anthracene and fluoranthene kinetics curves on a molar basis.**

**
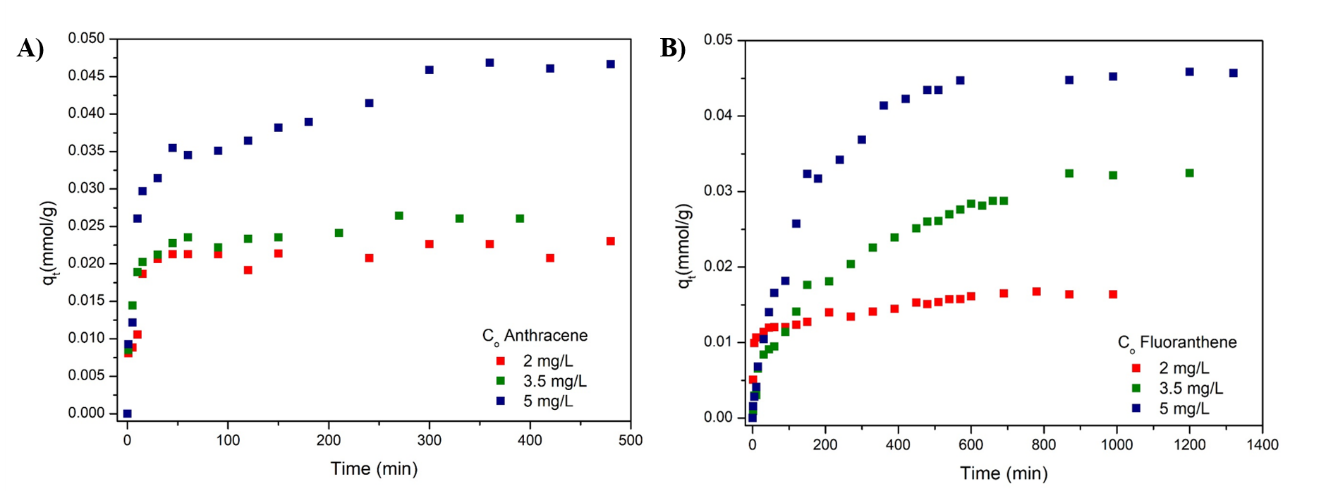
**

- **Equilibrium isotherms**

**Figure S13.** Adjustments of Langmuir, Freundlich and Sips isotherm models for anthracene adsorption at A) 25°C, B) 35°C and C) 45°C.

**
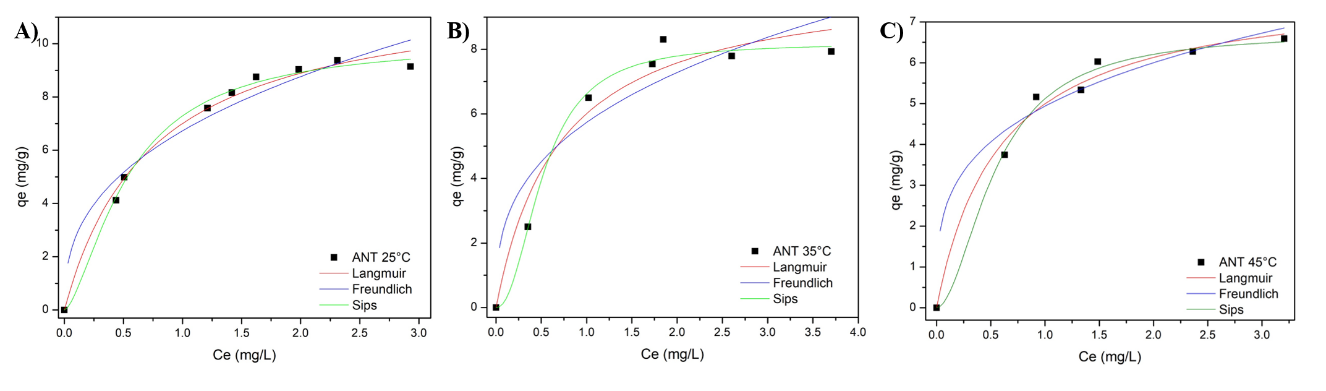
**

**Figure S14.** Adjustments of Langmuir, Freundlich and Sips isotherm models for fluoranthene adsorption at A) 25°C, B) 35°C and C) 45°C.

**
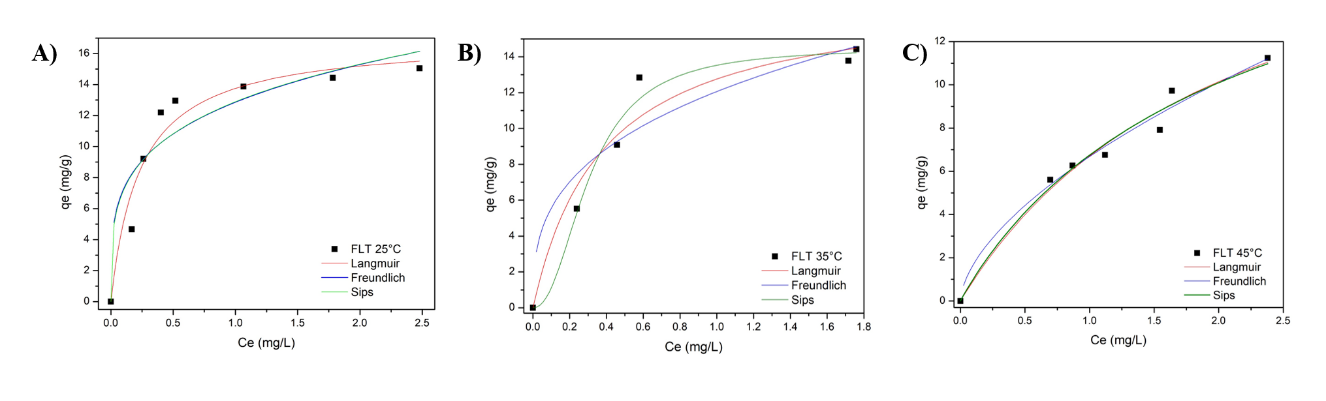
**

**Table S6** Effective diffusion rates determined by Boyd, Linear driving force, and Fickian diffusion models for anthracene and fluoranthene adsorption onto m-rGO@CS (C_o_=2mg/L, 25°C)

| **Compound** | **D_f_ (m^2^/min)** | | |
| --- | --- | --- | --- |
|  | **Boyd** | **LDF** | **FD** |
| Anthracene | 8.1x10^-9^ | 1.2x10^-9^ | 7.5x10^-10^ |
| Fluoranthene | 6.8x10^-9^ | 3.9x10^-11^ | 3.9x10^-12^ |

- Additional Thermodynamic analysis

To further substantiate the thermodynamic analysis discussed in the main manuscript, the thermodynamic parameters were additionally estimated based on the equilibrium constants obtained from the Langmuir isothermal model (Table S7). For fluoranthene adsorption, the calculated parameters exhibited values of the same order of magnitude as those previously reported, thereby reinforcing the reliability and internal consistency of the thermodynamic evaluation. These findings confirm that fluoranthene adsorption onto m-rGO@CS beads occurs spontaneously and is an exothermic process. Moreover, the positive ΔS values indicate a degree of structural reorganization within the system. In the case of anthracene adsorption, the estimated thermodynamic parameters revealed a more noticeable variation compared to the values reported in the main text. Nonetheless, the overall interpretation remains consistent: the adsorption process is exothermic, feasible, and spontaneous, accompanied by an increase in the degrees of freedom of the adsorbed molecules at the adsorbent–solution interface. It should be emphasized, however, that the parameters estimated from the equilibrium constants (Kc) derived using the Langmuir model exhibited weak coefficients of determination, indicating a weaker correlation with the experimental data. Therefore, these results are presented here solely to corroborate and reinforce the trends and conclusions discussed in the main manuscript, rather than as a primary basis for thermodynamic interpretation.

**Table S7** Thermodynamic parameters of anthracene and fluoranthene adsorption onto m-rGO@CS.

| **Compound** | **Adsorbent** | **Temperature (K)** | **ΔH (kJ/mol)** | **ΔS (J/mol.K)** | **ΔG (kJ/mol)** |
| --- | --- | --- | --- | --- | --- |
| Anthracene | m-rGO@CS | 298 | −1.12 | 96.67 | −28.00 |
|  |  | 308 |  |  | −30.90 |
|  |  | 318 |  |  | −31.06 |
| Fluoranthene | m-rGO@CS | 298 | −13.85 | 57.10 | −29.72 |
|  |  | 308 |  |  | −31.43 |
|  |  | 318 |  |  | −32.01 |

**Table S8** Isosteric heat values of anthracene and fluoranthene adsorption onto m-rGO@CS

| **q_e_ (mg/g)** | **ΔH_st_ (kJ/mol)** | | | |
| --- | --- | --- | --- | --- |
|  | **ANT** | **R^2^** | **FLT** | **R^2^** |
| 0.5 | −14.564 | 0.998 | −77.880 | 0.896 |
| 1.0 | −11.736 | 0.842 | −76.678 | 0.898 |
| 1.5 | −10.323 | 0.626 | −75.438 | 0.900 |

- **Isosteric enthalpy of adsorption**

**Figure S15.** Isosteres obtained for the adsorption of anthracene and fluoranthene onto m-rGO/CS beads.

**
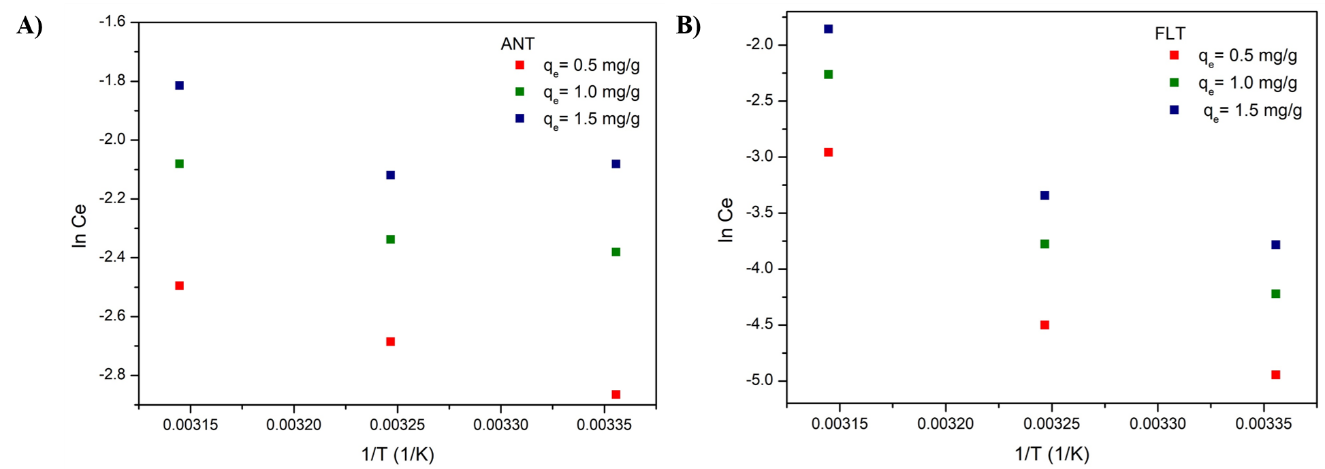
**

- **XPS analysis**

**Figure S16.** XPS A) N1s and B) Fe 2p high-resolution spectra for m-rGO@CS.

**
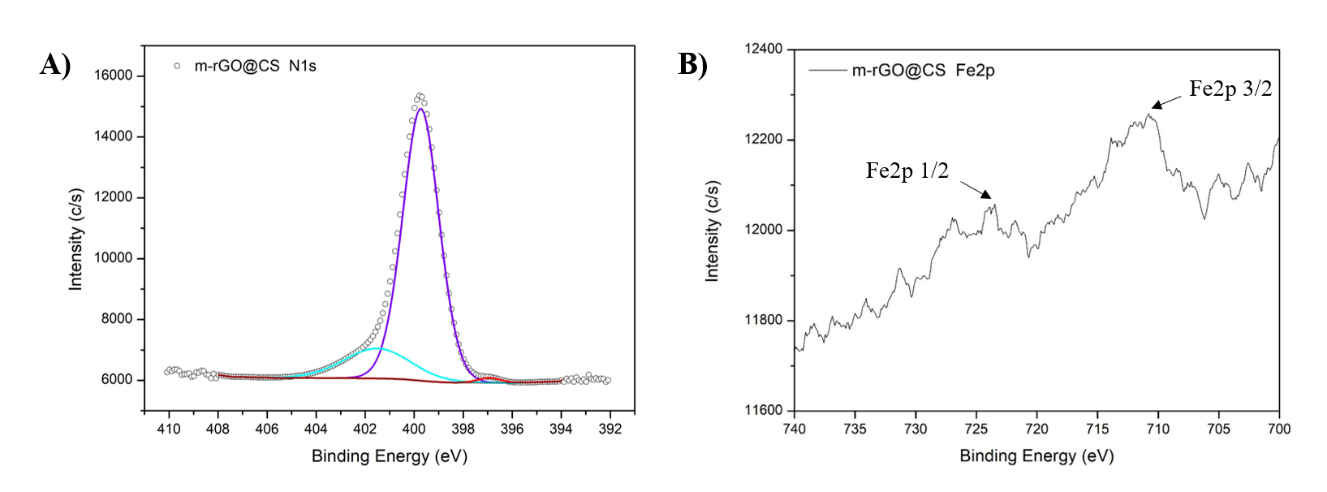
**

**Figure S17.** XPS A) N1s and B) Fe 2p high resolution spectra for m-rGO@CS+ANT

**
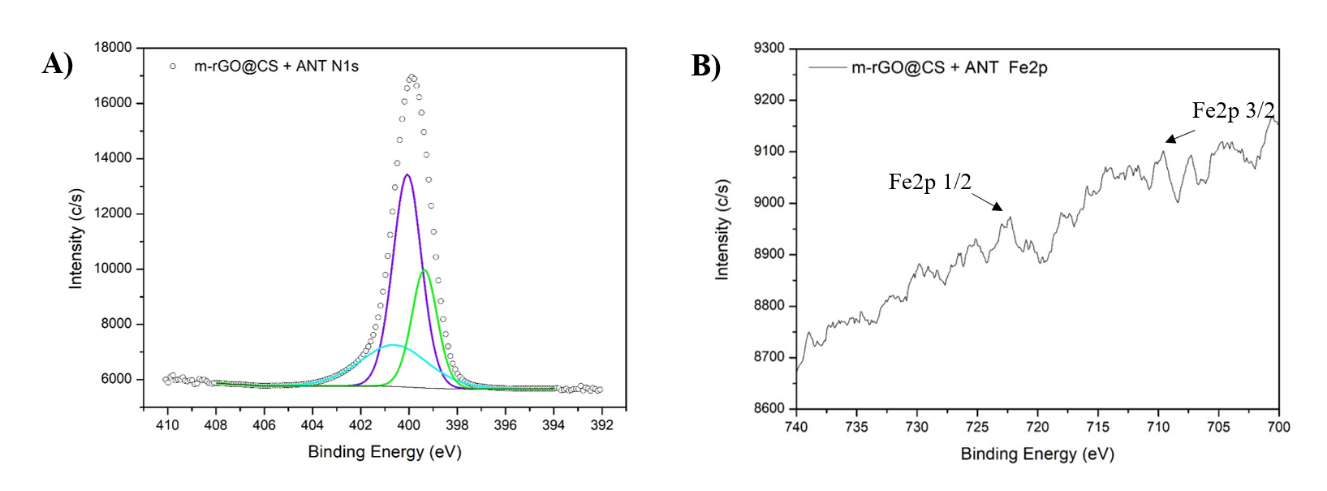
**

**Figure S18.** XPS A) N1s and B) Fe 2p high resolution spectra for m-rGO@CS+FLT

**
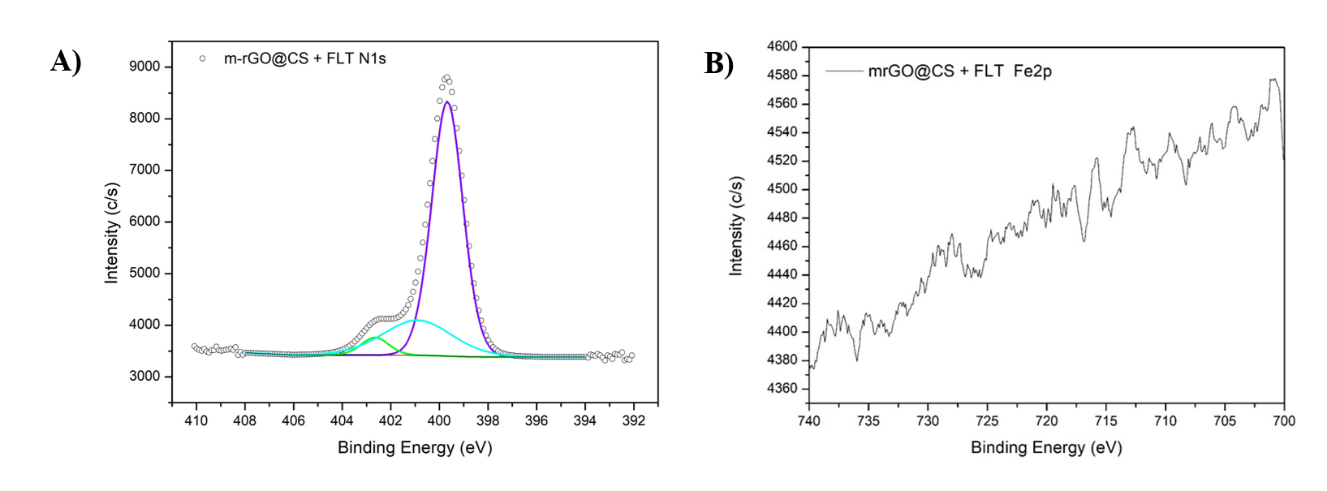
**

**Figure S19.** Wide-scan spectra of m-rGO@CS

**
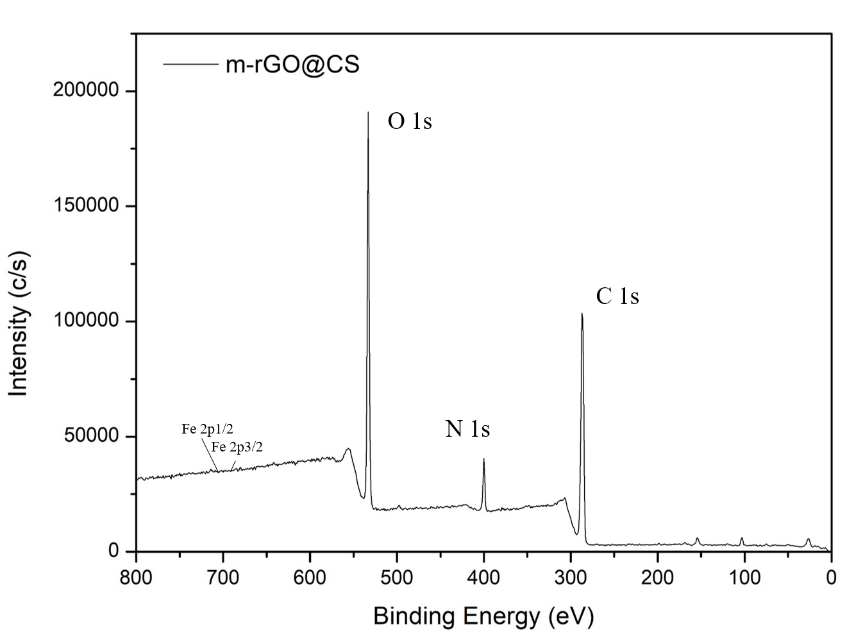
**

**Figure S20.** Wide-scan spectra of m-rGO@CS+ANT

**
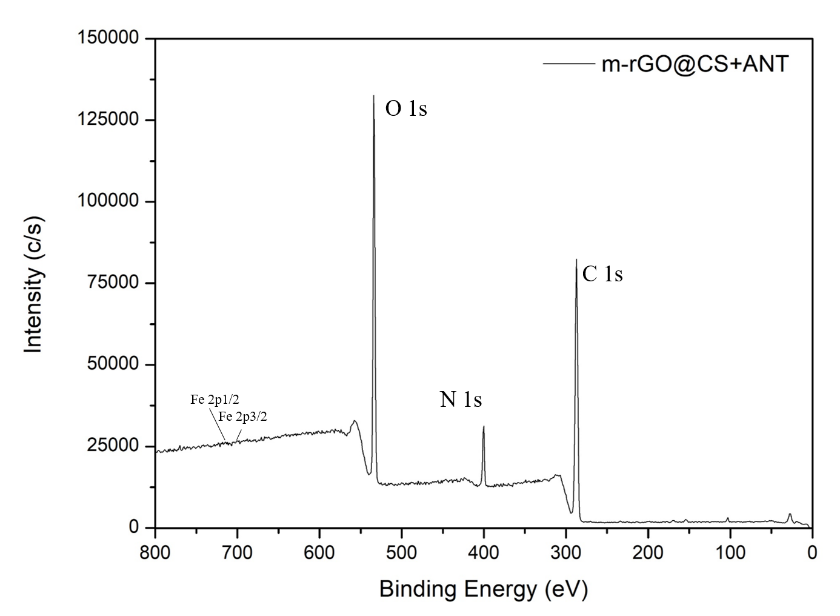
**

**Figure S21.** Wide-scan spectra of m-rGO@CS+FLT.

**
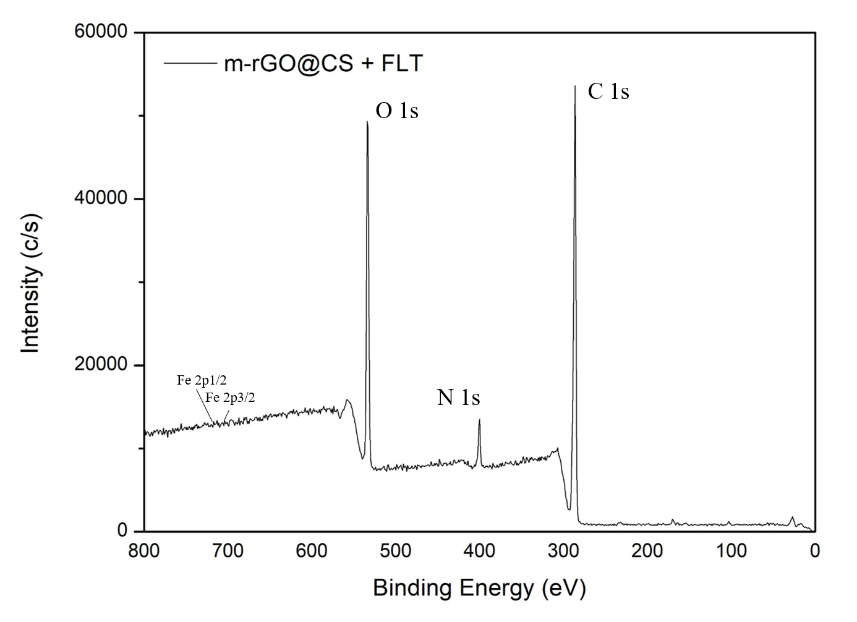
**

**Fig. S22** Simplified batch adsorption design for A) anthracene and B) fluoranthene uptake by m-rGO@CS

- **BET anlysys**

**Table S9.** Nitrogen adsorption–desorption parameters.

| **Parameters** | **Values** |
| --- | --- |
| BET surface area | 0.1644 m²/g |
| BET C constant | 4.09 |
| Monolayer capacity (Qm) | 0.0378 cm³/g STP |
| Single-point total pore volume (P/P₀ ≈ 0.99) | 1.72 × 10⁻⁴ cm³/g |
| BJH adsorption pore volume | 1.42 × 10⁻⁴ cm³/g |
| BJH desorption pore volume | 1.62 × 10⁻⁴ cm³/g |
| BJH adsorption avg. pore diameter | 74.9 Å (7.5 nm) |
| BJH desorption avg. pore diameter | 43.3 Å (4.3 nm) |
| Isotherm type | Type IV, with H3 hysteresis loop |

**Figure S23.** BET Nitogen adsorption-desorption isotherm**.**


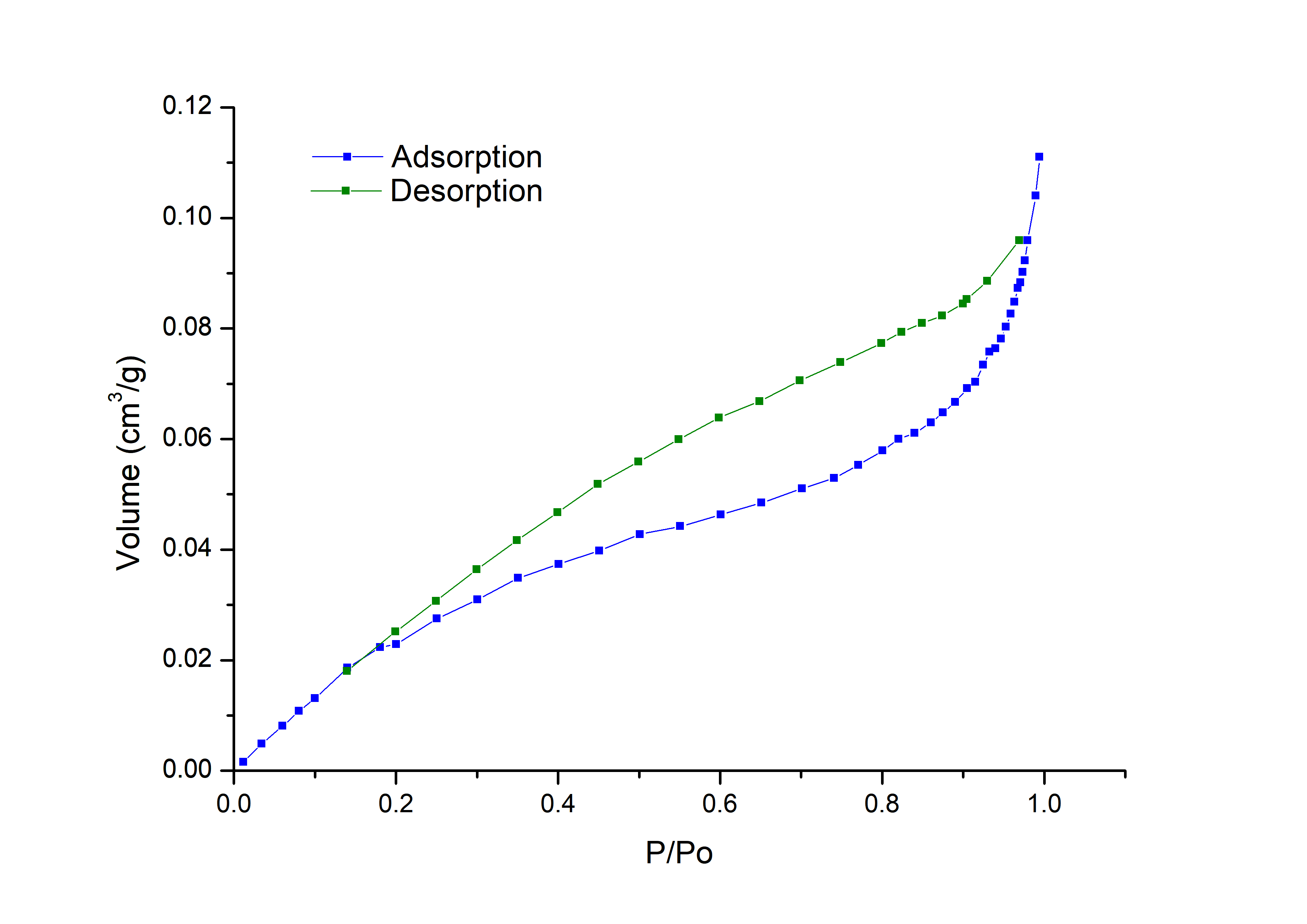


**References**

Boyd GE, Adamson AW, Myers LS (1947) The Exchange Adsorption of Ions from Aqueous Solutions by Organic Zeolites. II. Kinetics. J Am Chem Soc 69:2836–2848. https://doi.org/10.1021/ja01203a066

Freundlich H (1906) Over the adsorption in solution. J Phys Chem 57:385-470

Ho YS, McKay G (2000) The kinetics of sorption of divalent metal ions onto sphagnum moss peat. Water Res 34:735–742. https://doi.org/10.1016/S0043-1354(99)00232-8

Lagergren KS (1898) About the Theory of So-called Adsorption of Soluble Substances. Sven Vetenskapsakad Handingarl 24:1–39

Langmuir I (1918) The adsorption of gases on plane surfaces of glass, mica and platinum. J Am Chem Soc 40:1361–1403. https://doi.org/10.1021/ja02242a004

Puranik PR, Modak JM, Paknikar KM (1999) Comparative study of the mass transfer kinetics of metal biosorption by microbial biomass. Hydrometallurgy 52:189–197. https://doi.org/10.1016/S0304-386X(99)00017-1

Sips R (1948) On the structure of a catalyst surface. J Chem Phys 16:490–495. https://doi.org/10.1063/1.1746922

Toth J (1971) State equations of the solid-gas interface Layers. Acta Chim Acad Sci Hung 311–317

Weber WJ, Morris JC (1963) Kinetics of Adsorption on Carbon from Solution. J Sanit Eng Div 89:
